# Functional characterization of the PHT1 family transporters of foxtail millet with development of a novel *Agrobacterium*-mediated transformation procedure

**DOI:** 10.1038/s41598-017-14447-0

**Published:** 2017-10-25

**Authors:** S. Antony Ceasar, Alison Baker, S. Ignacimuthu

**Affiliations:** 1 Centre for Plant Sciences and School of Molecular and Cellular Biology, Faculty of Biological Sciences, University of Leeds, Leeds, LS2 9JT UK; 2 Division of Plant Biotechnology, Entomology Research Institute, Loyola College, Chennai, 600034 India

## Abstract

Phosphate is an essential nutrient for plant growth and is acquired from the environment and distributed within the plant in part through the action of phosphate transporters of the PHT1 family. Foxtail millet (*Setaria italica*) is an orphan crop essential to the food security of many small farmers in Asia and Africa and is a model system for other millets. A novel *Agrobacterium*-mediated transformation and direct plant regeneration procedure was developed from shoot apex explants and used to downregulate expression of 3 members of the PHT1 phosphate transporter family *SiPHT1*;*2 SiPHT1*;*3* and *SiPHT1*;*4*. Transformants were recovered with close to 10% efficiency. The downregulation of individual transporters was confirmed by RT-PCR. Downregulation of individual transporters significantly reduced the total and inorganic P contents in shoot and root tissues and increased the number of lateral roots and root hairs showing they have non-redundant roles. Downregulation of *SiPHT1*;*2* had the strongest effect on total and inorganic P in shoot and root tissues. Complementation experiments in *S*. *cerevisiae* provide evidence for the ability of *SiPHT1*;*1*, *1*;*2*, *1*;*3*, *1*;*7* and *1*;*8* to function as high affinity Pi transporters. This work will aid development of improved millet varieties for global food security.

## Introduction

Phosphorous (P) is an important macronutrient for all living organisms as it plays several important biochemical functions. P participates in the structure of nucleotides and phospholipids, and it is also important for many biochemical functions including photosynthesis and respiration. P is important for plant growth and P deficiency affects the growth and yield of several crop plants^1^. Plants absorb P from soil solution as inorganic phosphate (Pi), the process is influenced by the soil pH^2^.

High reactivity with metal cations and very low mobility of Pi make it highly inaccessible to plants. Hence, organic or synthetic P fertilizers are applied to improve crop yields. Reserves of rock phosphate for the production of inorganic phosphate fertilizers are predicted to be exhausted in next 2 centuries or so^3,4^. Further, much of the applied phosphate is also wasted, resulting in environmental damage^1^. So it is imperative to find ways to improve the Pi uptake efficiency of crop plants for sustainable food production.

The principal means of entry of Pi from the soil to plant is through the plasma membrane located PHOSPHATE TRANSPORTER 1 (PHT1) family^5^. Each plant has several members of PHT1 transporters which are found to play key roles not only in P acquisition but also in root to shoot transport and remobilization from mature to young tissues. Since their discovery in *Arabidopsis thaliana* in 1996 ^6^, members of these transporters have been characterized in several plants including in rice, wheat, maize, barley and sorghum^1,5^. Many of the PHT1 members have been found to be induced by low Pi stress in root tissues. Their functions were characterized by heterologous expression in *Saccharomyces cerevisiae* Pi transporter deficient mutants and Xenopus oocytes. PHT1 transporters are found to have 12 transmembrane segments and belong to the Major Facilitator Superfamily (MFS) of transporters^5,7^.

Millets are important cereals used as a food and feed in developing countries of Asia and Africa. Foxtail millet (*Setaria italica*) is one of the important millets mostly cultivated in the semi-arid regions of Asia and Africa and has been used as an excellent model species for various genetic studies^8,9^. Together with its wild relative green foxtail (*Setaria viridis*), it is the only millet with its whole genome sequenced^9,10^. While the genome sequencing of other millets like finger millet and pearl millet are still in progress due to their larger genome size^11^, foxtail millet has been considered as an ideal millet to study the functions of PHT1 transporters. Although millets are important cereal crops in less developed countries, they have not been paid much attention for genetic transformation studies^12^. Even only a few reports are available on the *Agrobacterium*-mediated transformation of foxtail millet^13–15^. However, in all these reports immature inflorescence was used as initial explants and callus mediated regeneration was adopted to recover the transgenic plants. The callus mediated regeneration is a time consuming process and may also induce soma clonal variations. Hence, development of rapid regeneration protocol is needed for this important model crop following an efficient *Agrobacterium*-mediated transformation.

In a previous study, we have analysed the expression patterns of *12 PHT1* transporters of foxtail millet (*SiPHT1*;*1* to *SiPHT1*;*12*) as a function of external Pi concentration and in response to colonisation of arbuscular mycorrhizal fungus (AMF) *Funneliformis mosseae* ^8^. The transporter *SiPHT1*;*2* has been found to express in all tissues and at all stages of growth tested while *SiPHT1*;*4* was found to be induced by low Pi stress in root tissues. Many transporters were also induced in 15 days old shoot tissues under low Pi condition. Transporters *SiPHT1*;*8* and *SiPHT1*;*9* have been found to be induced by AMF colonisation in root tissues^8^. However, functions of these transporters have not yet been studied.

In this article we report a novel *Agrobacterium*-mediated transformation system using shoot apex explants with a direct plant regeneration procedure. Yeast complementation studies of some of these transporters was performed in a *Saccharomyces cerevisiae pho84* mutant and the *in planta* functions of 3 transporters (SiPHT1;2, 1;3 and 1;4) studied by downregulation through RNA interference (RNAi). This study will help to understand the role of PHT1 transporters on Pi transport in foxtail millet with a view to improve Pi uptake and efficient utilization under low Pi stress conditions. The efficient *Agrobacterium*-mediated transformation system reported in this study may be useful for other millets and closely related wild relative like green foxtail (*Setaria viridis*).

## Results

### PHT1 transporters of foxtail millet can complement the low phosphate growth deficiency of a PHO84 deficient yeast mutant

**Figure 1:**
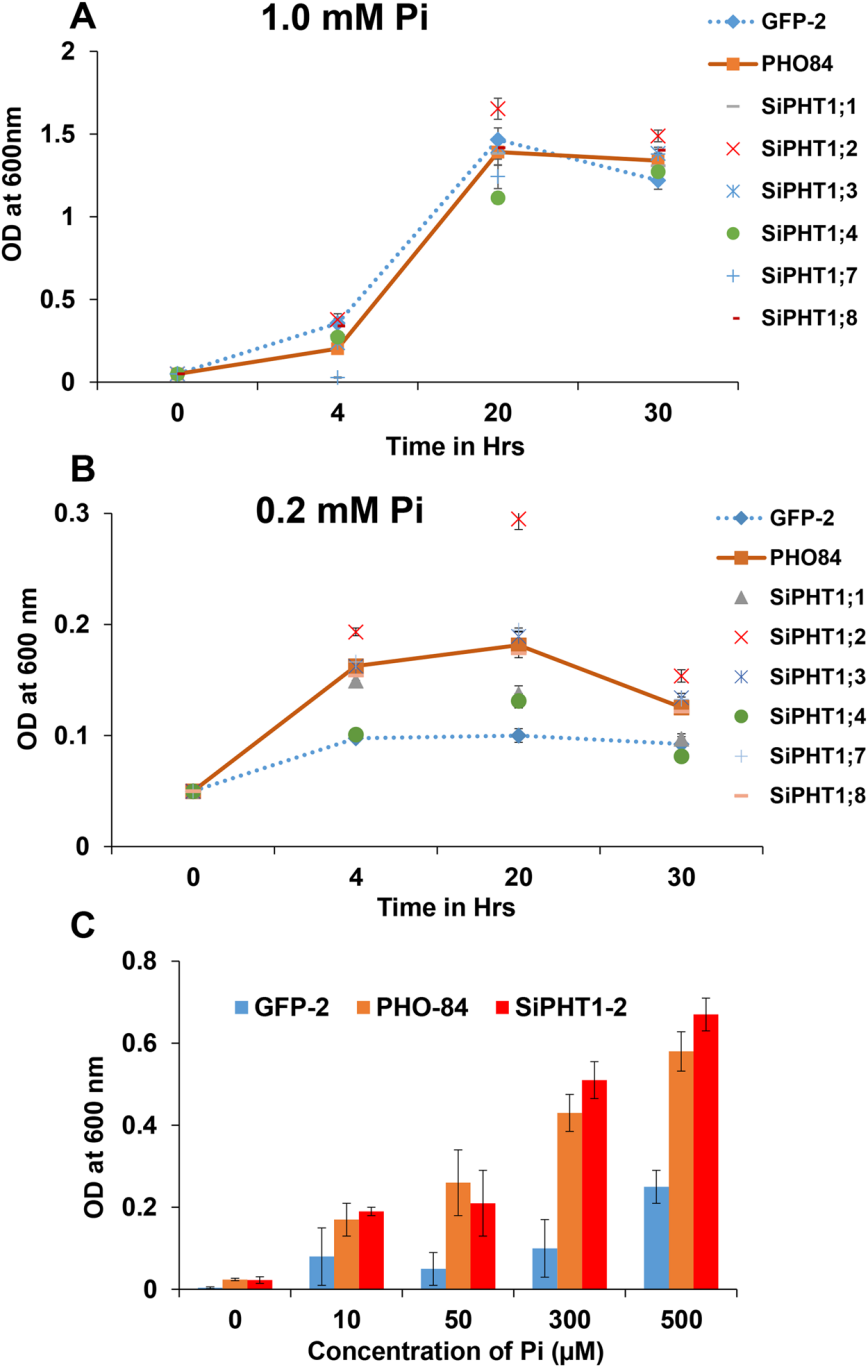
Complementation of *S*. *cerevisiae pho84* mutant with foxtail millet PHT1 transporters. Growth curves of *S. cerevisiae* *pho84Δ* mutant transformed with the indicated foxtail millet SiPHT1 transporters, empty vector (GFP-2 dotted line) and *S*. *cerevisiae* PHO84 (solid line) under 1.0 mM Pi (**A**) and 0.2 mM Pi Pi (**B**). Assay of growth of *pho84Δ* mutant cells transformed with *SiPHT1*;*2*, *ScPHO84* or empty vector (GFP-2) in different concentrations of Pi (**C**). The OD_600_ was measured after 20 hrs of growth. All the values are expressed as mean ± SD of 3 replicates and 2 repeats. The initial OD_600_ cultures were adjusted to have the starting of 0.05.

To determine whether the SiPHT1 family encodes functional phosphate transporters, six members (SiPHT1;1, 1;2, 1;3 1;4 1;7 and 1;8) were tested for the ability to complement the growth of a *S*. *cerevisiae Δpho84* mutant under high (1.0 mM) and low (0.2 mM) Pi. The cells were initially grown on 1 mM Pi with the addition of 2% galactose for the induction of expression of the transporter genes. On 1 mM Pi, all strains, including the empty vector transformed (Fig. 1A, GFP2 dotted line) showed similar growth curves to the positive control (Fig. 1A, PHO84 solid line) reaching stationary phase by 20 h with an OD_600_ between 1 and 1.5, showing that all strains were viable (Fig. 1A). When the experiment was repeated using 0.2 mM Pi, as expected, the empty vector control (Fig. 1B, GFP2 dotted line) barely grew. In contrast the mutant transformed with endogenous *PHO84* gene (Fig. 1B, solid line) grew better than the negative control, reaching an OD_600_ of 0.18 after 20 h. With the exception of *SiPHT1*;*4*, the cells transformed with the SiPHT1 transporters grew similarly to, or in the case of the *SiPHT1*;*2* transporter even better than, those transformed with the endogenous *PHO84* transporter (Fig. 1B).

Cells transformed with empty vector (GFP2-negative control), native *PHO84* (PHO84 positive control) and *SiPHT1*;*2* were further examined by growing them in different concentrations of Pi (10, 50, 300 and 500 µM Pi) (Fig. 1C). Cells transformed with either of *PHO84* or *SiPHT1*;*2* grew much better than the negative control, even at 10 µM Pi, with the difference being more marked at 50 and 300 µM. At 500 µM, the negative control could grow but still to a much lesser extent than the positive control and *SiPHT1*;*2*, whereas in 1 mM Pi the positive and negative controls grew similarly (Fig. 1A). Thus all the transporters tested with the possible exception of *SiPHT1*;*4* could complement the *S*. *cerevisiae Δpho84* mutant for growth on 0.2 mM Pi.

### Development of a novel direct plant regeneration and *Agrobacterium*-mediated transformation systems

**Figure 2:**
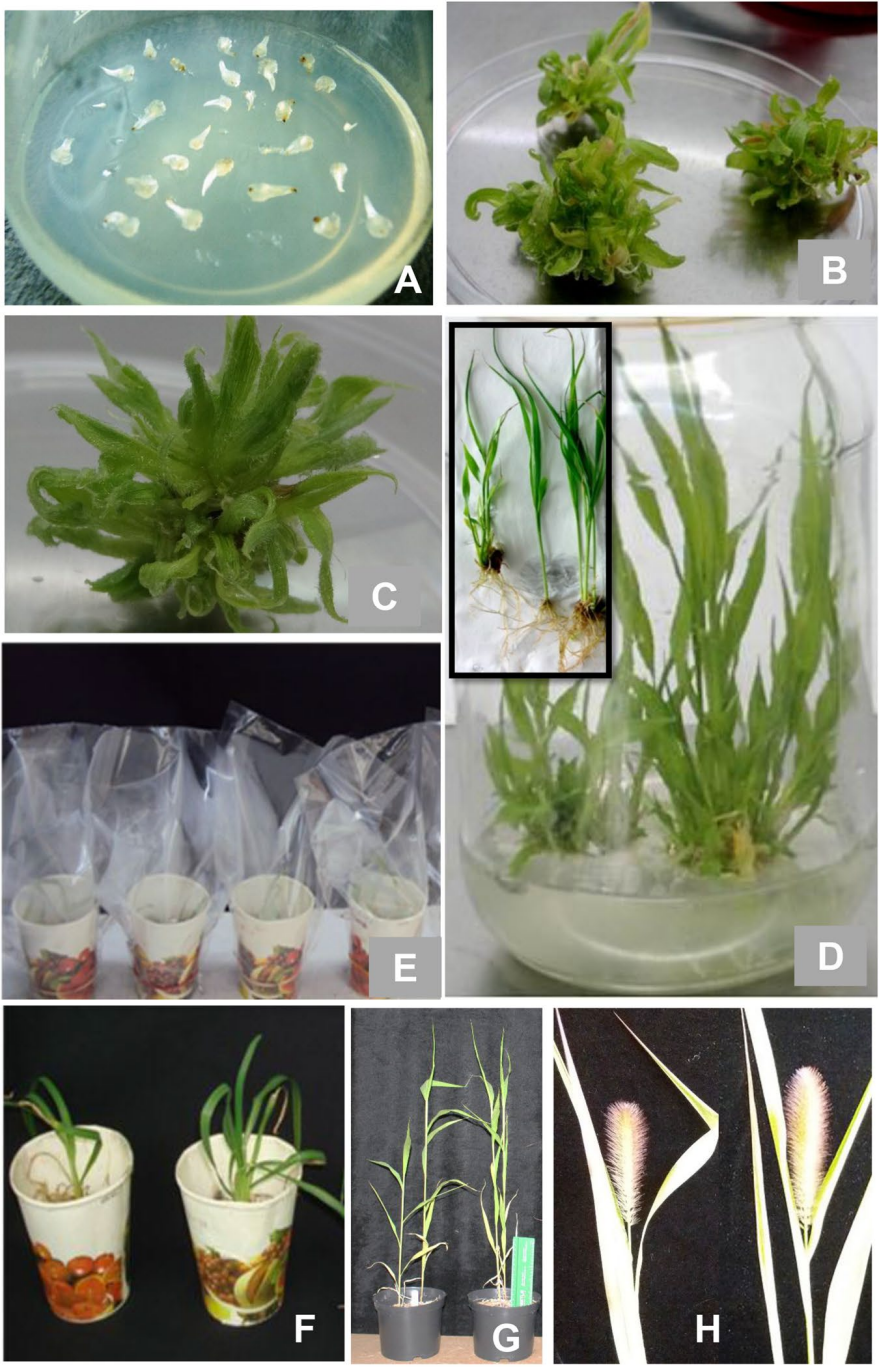
Direct plant regeneration from shoot apex explants foxtail millet genotype Maxima. Inoculation of shoot apex explants on MS medium containing BAP, after 3 days of incubation in the light (**A**), induction of multiple shoots on shoot induction medium (SIM) containing MS salts and 0.5 mg/L BAP, after 2 weeks of incubation in the light (**B**), Elongation of shoots in shoot elongation medium (SEM) consisting of MS salts, after one week of incubation in the light (**C**), rooting of shoots in the SEM after 3 weeks of incubation in the light (**D**). The inset figure shows the fully rooted plants removed from the vessels (**D**). Plants transferred from culture vessels to paper cups containing sterile vermiculite (**E**), paper cups were removed after 2 weeks (**F**), regenerated plants grown in green house, after 4 weeks of growth (**G**), seed setting in the *in vitro* regenerated plants (**H**).

Whilst heterologous complementation is a convenient test for functionality of phosphate transporters, to understand their roles within the physiology of the plant phosphate response requires manipulation of expression *in planta*. To this end an *Agrobacterium*-mediated transformation and novel direct regeneration protocol was developed for foxtail millet. Shoot apex explants were initially cultured on Murashige and Skoog (MS) medium containing various concentrations of cytokinins benzylamino purine (BAP), thidiazuron (TDZ) or kinetin (KN) at varied concentrations (Fig. 2A; Table 1). Multiple shoot induction was seen in all phytohormone containing media after 2 weeks of incubation, with significantly higher number of shoots per explant (28 ± 0.89) in the MS medium containing 0.5 mg/l BAP, after 4 weeks of incubation in the light (Table 1; Fig. 2B). Based on this study, MS medium containing 0.5 mg/l BAP was used in subsequent experiments as the shoot induction medium (SIM). The shoot clumps with multiple shoots obtained from MS medium containing 0.5 mg/l BAP were transferred onto the shoot elongation medium (SEM) containing MS salts alone devoid of phytohormones for shoot elongation. The shoot clumps cultured on the SEM elongated to produce the normal plants within 2 weeks (Fig. 2C). The shoots were very healthy and green in appearance. The plants were rooted in the same medium (SEM) after 3 weeks of incubation (Fig. 2D). After transfer to vermiculite and maintenance under polythene for a further 2 weeks for hardening (Fig. 2E and F), the plants were moved to the greenhouse with 100% survival rate and grown to maturity (Fig. 2G and H). The plants were grown up to seed setting and no variation in regenerated plants for growth, flowering and seed setting was observed when compared to the wild plants (non-tissue culture) grown from seeds.

**Table 1:** Effect of phytohormone on multiple shoot induction from shoot apex explants in foxtail millet genotype Maxima. Response was noted after 3 weeks of incubation at 25 ± 2 °C in light. *Values are expressed as mean ± SD of 3 replications and 3 repeats. Values followed by the same letter are not significantly different based on a t-test (P < 0.001).

Name of the phytohormone	Concentration (mg/l)	Percentage of explants responding	Number of shoots/explant*
KN	0.0	0.0	0
	0.5	36.5	16 ± 1.1 c
	1.0	28.4	13 ± 0.81 b
	1.5	19.7	9 ± 0.73 d
	2.0	13.5	4 ± 0.82 e
BAP	0.0	0.0	0
	**0.5**	**95.7**	**28 ± 0.89 a**
	1.0	81.8	23 ± 0.75 b
	1.5	38.6	15 ± 1.1 c
	2.0	32.5	7.0 ± 1.9 d
TDZ	0.0	0.0	0
	0.5	34.7	18 ± 0.75 b
	1.0	31.5	14 ± 0.81 c
	1.5	17.8	6 ± 0.19 d
	2.0	12.9	3 ± 0.54 e

**Figure 3:**
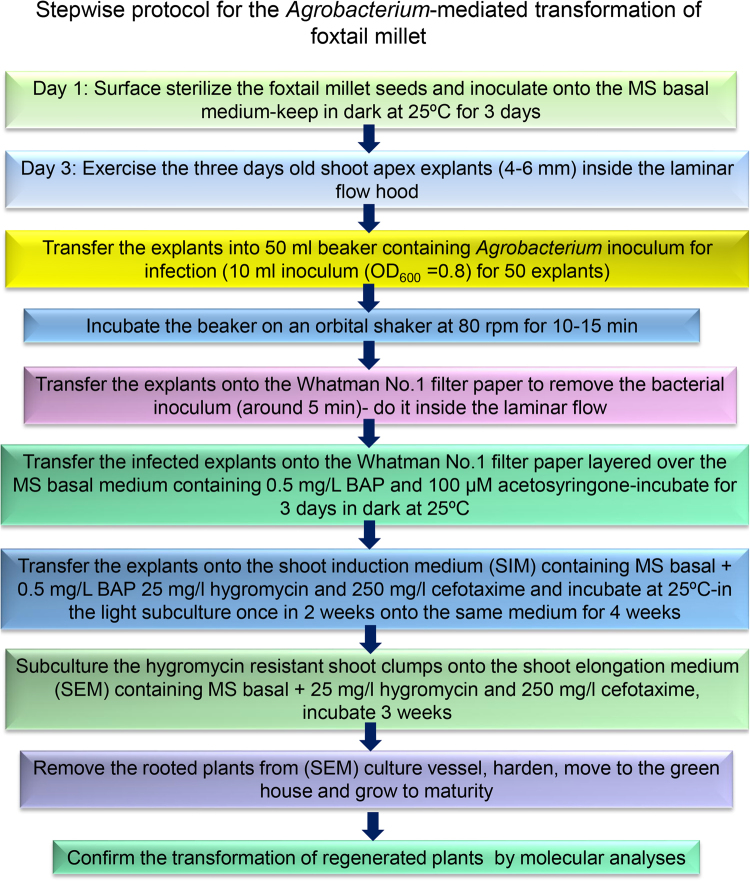
Stepwise protocol for *Agrobacterium*-mediated transformation of foxtail millet using shoot apex explants.

**Figure 4:**
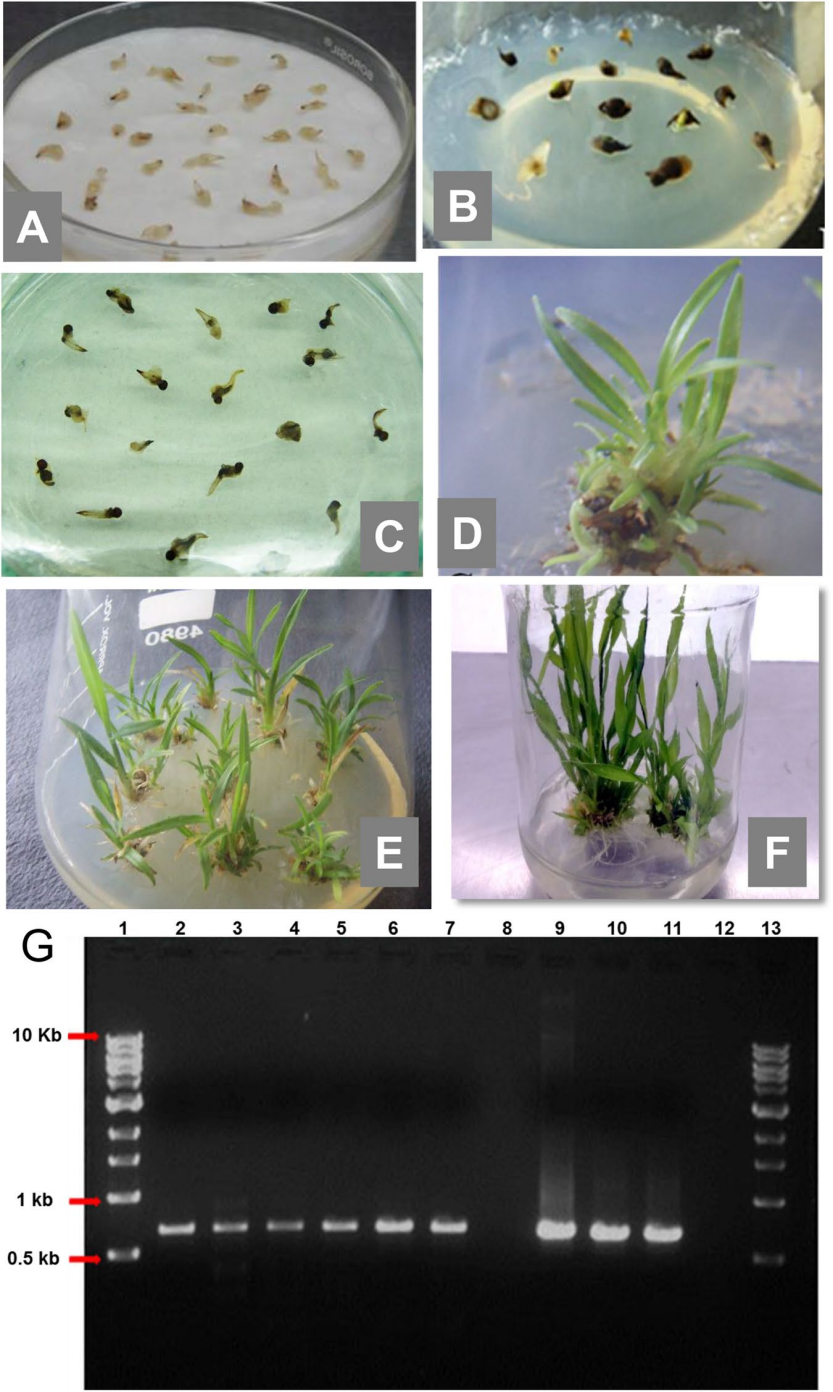
*Agrobacterium*-mediated transformation, regeneration using shoot apex explants and confirmation of transformation by PCR in foxtail millet genotype Maxima. Co-cultivation of shoot apex explants on sterile filter papers over MS medium containing 0.5 mg/L BAP and 100 µM acetosyringone after 3 days on incubation in the dark (**A**), selection of co-cultivated explants on the shoot induction (SIM) medium containing MS salts, 0.5 mg/L BAP, 25 mg/l hygromycin and 250 mg/l cefotaxime, after 2 weeks of incubation in the light (**B**). The production of hygromycin resistant shoots are visible (**B**), un-transformed (negative control) explants in the same SIM are dead after 2 weeks of incubation (**C**), production of hygromycin resistant shoots in the SIM with MS salts, 0.5 mg/L BAP, 25 mg/l hygromycin and 250 mg/l cefotaxime, after 4 weeks of incubation (**D**), elongation of shoots in the shoot elongation medium (SEM) medium containing MS salts, 25 mg/l hygromycin and 250 mg/l cefotaxime, after one week of incubation in the light (**E**), rooting of shoots in the same medium after 3 weeks of incubation in the light (**F**). Confirmation of transformation by detection of *hptII* gene in primary transformants (T0) by PCR (**H**). Lane 1 and 13 = 1 kb DNA ladder. Lane 2 and 3 = *SiPHT1*;*2 RNAi* lines. Lanes 4 and 5 = *SiPHT1*;*3 RNAi* lines. Lanes 6 and 7 = *SiPHT1*;*4 RNAi* lines. Lane 8 = negative control (genomic DNA of non-transformed plant). Lane 9 = pFGC-*SiPHT1*;*2-RNAi* plasmid (positive control). Lane 10 = pFGC*-SiPHT1*;*3-RNAi* plasmid (positive control). Lane 11 = pFGC-*SiPHT1*;*4-RNAi* plasmid (positive control). Lane 12 = Water control (no template DNA).

The direct plant regeneration system developed here was utilized for the subsequent *Agrobacterium*-mediated transformation of foxtail millet with RNAi vectors (*pFGC-SiPHT1*;*2*, *pFGC-SiPHT1*;*3* or *pFGC-SiPHT1*;*4*) (Supplementary Figures S1–S3) and recovery of stable transgenic plants. The stepwise protocol for *Agrobacterium*-mediated transformation is outlined in Fig. 3. Three days old shoot apex explants were found to be good source for the transformation of foxtail millet based on this study. The explants were infected with *Agrobacterium* for 10–15 mins. This time period was found to be sufficient for the attachment of *Agrobacterium* cells onto the shoot apex explants. Explants were co cultivated on filter paper without directly touching the media (to reduce moisture) for 3 d in dark (Fig. 4A), then transferred onto the SIM containing 25 mg/l hygromycin and 250 mg/l cefotaxime. Screening experiments with a range of hygromycin concentrations showed the explants were effectively killed at 20 mg/l or above (Supplementary Figure S4) so 25 mg/l was chosen for selection of transformed cells in this study. The explants produced hygromycin resistant tissue within a week of culture on the selection medium and some untransformed dead tissues were also observed (Fig. 4B). The negative control (untransformed) explants cultured on the selection medium did not produce any hygromycin resistant tissues (Fig. 4C). The hygromycin resistant shoot clumps sub-cultured onto the SEM containing 25 mg/l hygromycin and 250 mg/l cefotaxime also produced good response of shoot elongation after 2 weeks of incubation in light (Fig. 4D and E). The hygromycin resistant plants were rooted in the SEM containing 25 mg/l hygromycin and 250 mg/l cefotaxime (Fig. 4F). No *Agrobacterium* overgrowth or contamination during the selection and regeneration was observed. The plants were recovered and transferred to the greenhouse as mentioned in the plant regeneration results. The plants were then analysed for the confirmation of gene transfer mediated by *Agrobacterium*.

#### Confirmation of transformation by PCR

The genomic DNA was isolated from hygromycin resistant plants of RNAi lines grown in greenhouse (primary transformants, T0 lines). The presence of the transgene was confirmed using the hygromycin gene specific primers. For each RNAi line, around 35 transgenic plants were obtained from three replicates, each consisting of around 125 explants. The percentage transformation frequency of around 9.0% was obtained for each RNAi line (Table 2). The PCR amplification of 2 representative lines each of these constructs are shown in Fig. 4G. The expected band size of 683 bp is seen in positive control (plasmid; Fig. 4G, lanes 9–11) and for 2 lines each of RNAi transgenic plants (Fig. 4G, lanes 2–7). No band is seen for genomic DNA of non-transformed control plant (Fig. 4G, lane 8), confirming successful integration of the transgene from RNAi vector. These lines were used to develop the T1 progeny which were used for shoot and root morphology analysis, PHT1 transporter expression analysis and P uptake studies.

**Table 2:** Details of transgenic plants obtained and frequency of transformation for each RNAi line. For each treatment, three replicates were maintained. Values are expressed as the mean ± SD.

Name of the transgenic line	Number of explants used	Total number of transgenic plants obtained (confirmed by PCR)	Frequency of transformation (%)
*SiPHT1*;*2-RNAi*	126	35	9.2 ± 0.58
*SiPHT1*;*3-RNAi*	125	36	9.6 ± 0.37
*SiPHT1*;*4-RNAi*	121	33	9.0 ± 0.49

### SiPHT1;2, SiPHT1;3 and SiPHT1;4 transporters of foxtail millet have distinct, non-redundant roles in Pi transport *in planta*

**Figure 5:**
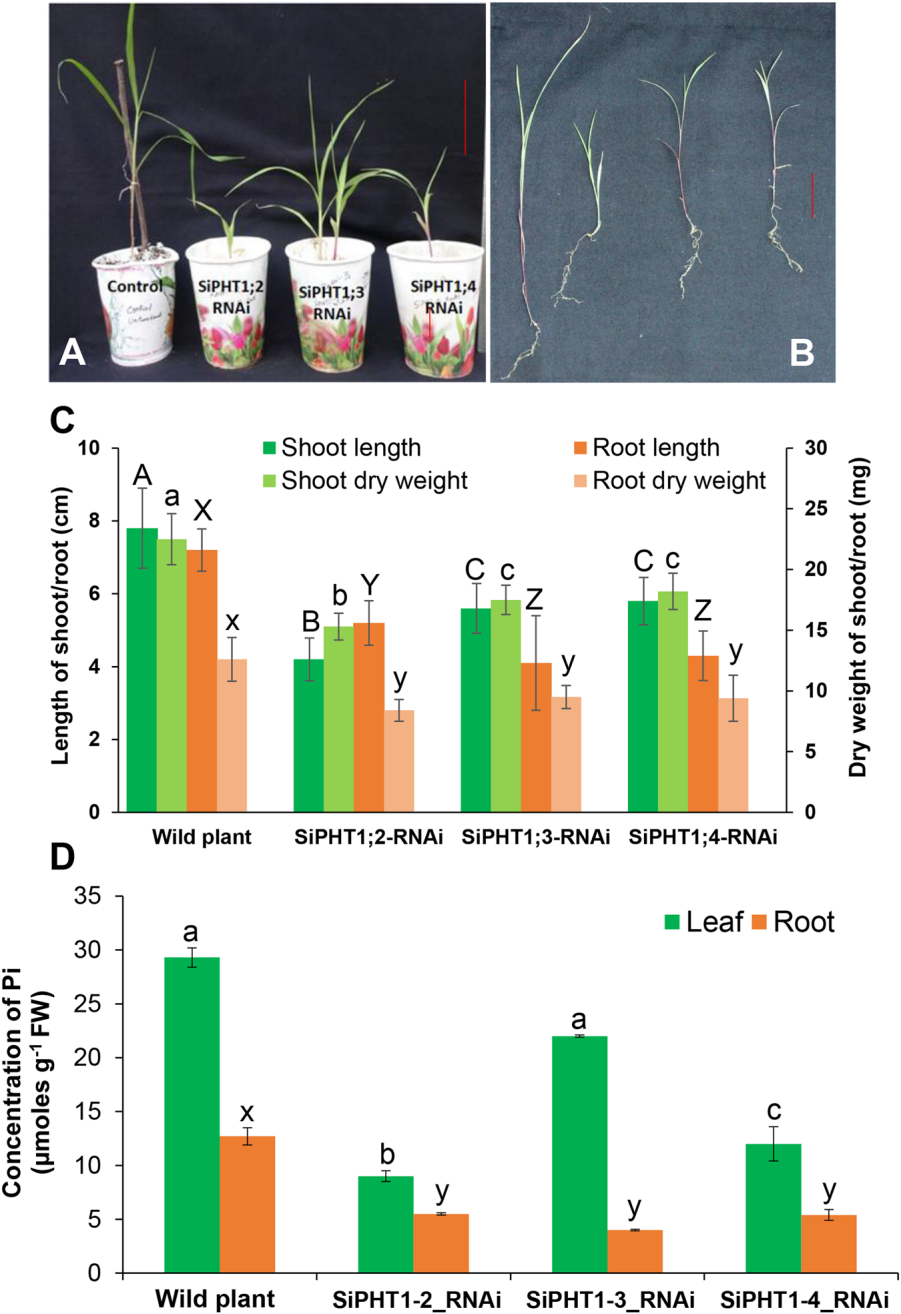
Phenotypic variation of foxtail millet seedlings of T1 lines after transformation with *SiPHT1*;*2*, *3* and *4 RNAi* vectors and analysis of Pi content. Three week old seedlings of the 3 RNAi lines and wild type plants grown under low Pi (10 µM) in paper cups containing perlite (**A**) (bar = 2 cm), seedlings removed from the paper cups showing shoot and root morphology, after 4 weeks of growth (**B**) (bar = 2 cm), shoot length and primary root length and dry weights of root and shoot of 4 weeks old seedlings of 3 RNAi lines and wild plants grown under low Pi (**C**), analysis of Pi content in shoot and root tissues of RNAi lines and wild plants grown under low Pi (**D**). In bar graphs, values are expressed as mean ± SD of 3 replicates (*n* = *3*). Values followed by the same letter are not significantly different (*P* > *0*.*001*) (separately for shoot length, root length, shoot dry weight, root dry weight and leaf and root Pi).

The phenotypic changes and Pi uptake abilities were assessed for T1 lines of RNAi plants grown under low Pi (10 µM). The RNAi lines showed retarded growth when compared to the wild type (control) plants (Fig. 5A and B). Among the 3 transgenic lines, *SiPHT1*;*2-RNAi* was much smaller when compared to other two lines (*SiPHT1*;*3-RNAi* and *SiPHT1*;*4-RNAi*). The photograph shows 2 week old plants grown under low Pi on perlite in the green house (Fig. 5A and B). The length of shoot and primary root and dry weights of shoot and root were determined (Fig. 5C). The RNAi lines showed significantly lower values for length and dry weights of shoot and root when compared to wild type non-transformed plants. *SiPHT1*;*2-RNAi* plants showed 50% reduction in shoot length and 26% reduction in root length when compared to the wild type plants. *SiPHT1*;*3-RNAi* and *SiPHT1*;*4-RNAi* transgenic plants showed only 30% reduction in shoot length and 15% reduction for primary root length when compared to control plants. Similarly significant reduction in dry weights of shoot and root were seen in RNAi plants when compared to wild plants (Fig. 5C), the *SiPHT1*;*2-RNAi* lines showed the greatest reduction in both shoot and root dry weights.

The three RNAi lines had lower Pi both in root and shoot tissues confirming the role of these transporters in both uptake of Pi from the soil and transport within the plant. The *SiPHT1*;*2-RNAi* lines showed a significantly lower level of Pi when compared to other two lines (*P* < *0*.*001*). These lines had only one third of the Pi in leaf samples and half of Pi in root samples when compared to control plants that were regenerated without transformation (Fig. 5D). *SiPHT1*;*4-RNAi* lines also showed significantly lower Pi in shoot tissues when compared to wild plants but significantly higher than those in *SiPHT1*;*2-RNAi* lines. The *SiPHT1*;*3-RNAi* lines had shoot Pi that were comparable to that of control plants (no significant difference) but it showed only one third of Pi in root tissues. The Pi content in root tissues of all 3 RNAi lines were also significantly lower when compared to the wild plants (Fig. 5D).

**Figure 6:**
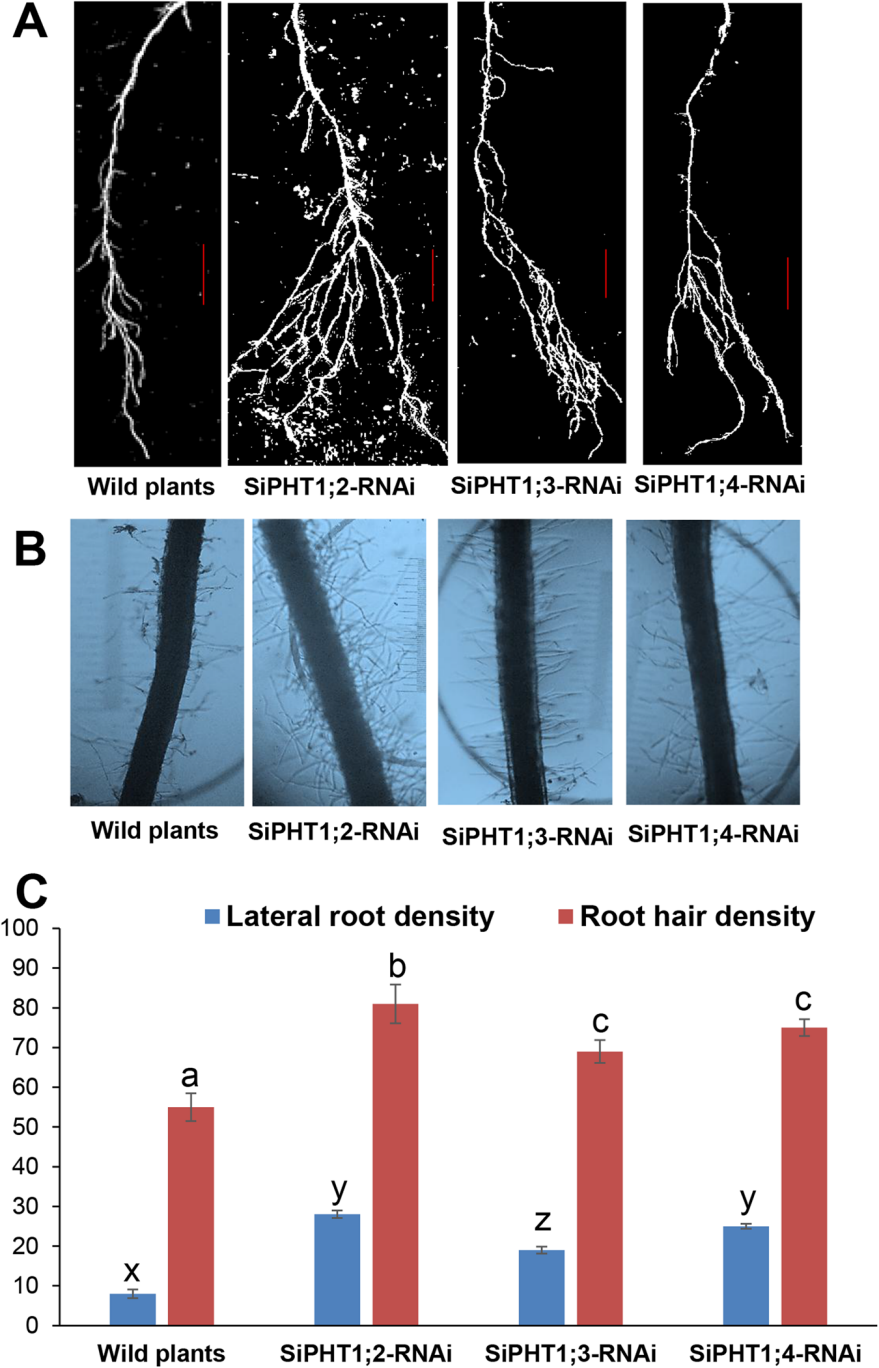
Analysis of root morphology in the 3 RNAi lines and wild plants grown under low Pi (10 µM) on hydroponics. Whole root images showing the lateral root formation in wild type and 3 RNAi lines grown after 4 weeks (**A**) (bar = 1 cm), root hair images of wild type plant and 3 RNAi lines after 4 weeks of growth (**B**), measurement of lateral root density per cm of root and root hair density per 10 µM of root in seedlings of wild type plants and 3 RNAi lines grown under low Pi on hydroponics for 4 weeks (**C**). Values are expressed as mean ± SD of 3 replicates (*n* = *3*). Values followed by the same letter are not significantly different (*P* > *0*.*001*) (separately for lateral root density shoot and root hair density).

To have tightly controlled supply of external Pi, further experiments were performed in hydroponics. Production of lateral roots and root hairs is an important adaptation to low P stress, therefore the lateral root density and root hair density were measured for wild type plants and in each RNAi line grown under low Pi in hydroponics. The images of lateral roots and root hairs of these plants are shown in Fig. 6A and B. The lateral root density (number per 1 cm length) was significantly higher in all RNAi lines when compared to the wild type plants under low Pi but the *SiPHT1*;*3-RNAi* line produced lateral root density higher than wild type plants but lower than the other 2 *RNAi* lines (Fig. 6C). Wild type and RNAi lines grown under low Pi produced root hairs with noticeable difference in densities (Fig. 6B). The highest root hair density was observed in *SiPHT1*;*2-RNAi* lines (Fig. 6A and B). *SiPHT1*;*3-RNAi* and *SiPHT1*;*4-RNAi* lines had higher root hair density than wild type plants but lower than the *SiPHT1*;*2-RNAi* lines.

### Expression pattern of PHT1 genes in T1 seedlings of RNAi lines

**Figure 7:**
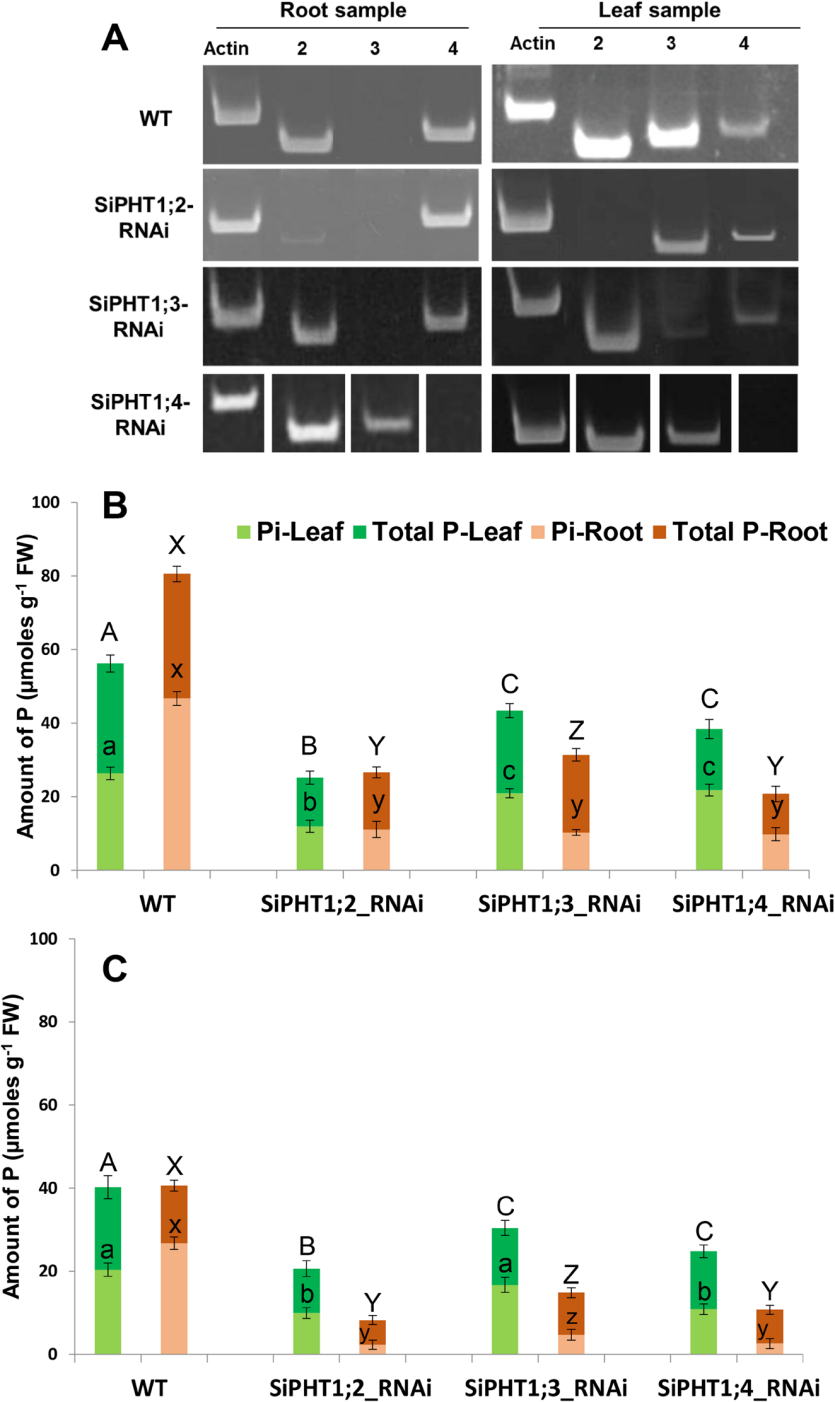
RT-PCR analysis and total and inorganic P content measurement in wild type plants and the 3 RNAi lines. (**A**) RT-PCR analysis of the 3 RNAi lines grown under low phosphorous (10 µM) using *Si-actin*, *SiPHT1*;*2*, *SiPHT1*;*3* and *SiPHT1*;*4* gene specific primers. WT = wild type plant (untransformed), *SiPHT1*;*2-RNAi* = transgenic plant downregulating *SiPHT1*;*2*, *SiPHT1*;*3-RNAi* = transgenic plant downregulating *SiPHT1*;*3*, *SiPHT1*;*4-RNAi* = transgenic plant downregulating *SiPHT1*;*4*. Lanes 2, 3 and 4 = amplification with *SiPHT1*;*2*, *SiPHT1*;*3* and *SiPHT1*;*4* gene specific primers respectively. Except *SiPHT1*;*4-RNAi* line, all the gels were cropped from various gels done at different time points. For *SiPHT1*;*4-RNAi* line, the individual lanes were from different gels which are indicated by delineating white lines. The individual gels were captured with different exposure times. T1 seedlings (4 weeks old) of RNAi lines grown under high Pi (**B**) and low Pi (**C**) were analysed for total and Pi contents. The total and Pi content of 2 weeks old (on hydroponics) seedlings were assayed using the same reported protocol^8^. The total height of the bars represents the total P. The lighter shading indicates that proportion of the total P that is Pi and the difference is the organic. Values were expressed as mean ± SD of 3 independent RNAi lines grown under low and high Pi. Values followed by the same letter are not significantly different (*P* > *0*.*001*).

The expression pattern of transporters *SiPHT1*;*2*, *1*;*3* and *1*;*4* were analysed by semi quantitative RT-PCR in all 3 RNAi lines grown on hydroponics under low Pi (10 µM) and compared to that of untransformed (wild type) plants (Fig. 7A). In total, 3 independent transformants were tested for each RNAi line for the reproducibility of results. The root and shoot samples of untransformed (wild type) plants produced the same pattern of expression for these 3 transporters as previously reported^8^. Transporters *SiPHT1*;*2* and *1*;*4* were found to be expressed in root tissues and transporters *SiPHT1*;*2* and *1*;*3* were expressed in leaf tissues. However, a little expression of isoform *SiPHT1*;*4* was seen in the leaf sample. The *SiPHT1*;*2-RNAi* line showed strongly reduced levels of *SiPHT1*;*2* transcript in root and leaf tissues. No band was seen corresponding to this gene specific RT-PCR product in leaf sample and only a very faint band was seen in root sample (Fig. 7A). Expression of the other 2 genes are not obviously altered in the *SiPHT1*;*2-RNAi* line. In the *SiPHT1*;*3-RNAi* line, *SiPHT1*;*3* gene specific transcripts were not detected in the root sample and only a very faint band is seen in leaf sample of this transgenic line. The expression patterns of the other genes remain unchanged. This result also confirmed that the RNAi construct designed specifically targeted *SiPHT1*;*3* and downregulated its expression. Bands corresponding to the *SiPHT1*;*4* transcript were not seen in either root or leaf tissue of the *SiPHT1*;*4-RNAi* line confirming the downregulation of this gene. A faint band was seen in leaf tissues of all other samples but even this band is also not seen in the leaf sample of *SiPHT1*;*4-RNAi* line. However downregulation *SiPHT1*;*4* resulted in upregulation of expression of *SiPHT1*;*3* in root samples.

### Total and inorganic P contents were reduced in all *SiPHT1 RNAi* lines

Total and inorganic P contents of leaf and root tissues were analysed in T1 progenies of RNAi lines grown hydroponically under 300 µM (Fig. 7B) and 10 µM Pi (Fig. 7C) respectively. In Fig. 7B and C, the height of the bar represents total P, the lighter shaded area within each bar the proportion of the total P that is inorganic P (Pi). The root samples of wild type plants had higher levels of both total and inorganic P than leaves under 300 µM (Fig. 7B). However, RNAi lines showed significantly lower levels of total and inorganic P in both root and leaf tissues under both high and low P conditions (Fig. 7B and C). In the *SiPHT1*;*2-RNAi* line grown on 300 µM Pi, both total and inorganic P contents were reduced by around 53% in leaf tissues when compared to wild type plants. In root tissues of this RNA lines, the total P content was reduced by 54% and 76% reduction of Pi compared to wild type plants (Fig. 7B). The *SiPHT1*;*3-RNAi* lines showed 24% and 20% reduction in total and inorganic P respectively in leaf samples. However the root samples *SiPHT1*;*3-RNAi* lines showed lower levels of both total and inorganic P than leaf samples; 37% reduction in total P and 78% reduction in Pi was seen in root tissues of these RNAi lines. *SiPHT1*;*4-RNAi* lines showed moderate reduction (44%) in total P content and with slightly lower levels of Pi (17%) than wild plants in leaf tissues. However, both total and inorganic P contents of root tissues of the same RNAi lines showed much lower levels (67% reduction in total P and 79% reduction in Pi) than wild plants.

The RNAi lines and wild type plants grown in 10 µM Pi showed lower levels for both total and inorganic P contents in root and leaf tissues than those grown under 300 µM Pi. The total P content in leaf and root tissues of wild type plants was almost the same with slightly higher levels of Pi in root than in leaf tissues. All 3 RNAi lines had significantly lower levels of both total and inorganic P content in root and leaf tissues (Fig. 6C). All 3 RNAi lines showed very low levels of total and inorganic P in root tissues compared to leaf tissues. *SiPHT1*;*2-RNAi* lines had significantly lower levels of total and inorganic P in leaf tissues than wild plants with 49% reduction when compared to wild plants, the same RNAi lines showed 57% reduction of total P and 91% reduction of Pi in root tissues. The total P content in leaf tissues of *SiPHT1*;*3-RNAi* and *SiPHT1*;*4-RNAi* lines were higher than *SiPHT1*;*2-RNAi* lines but lower than wild type plants with significantly different values (*P* > *0*.*001*). In root tissues, the *SiPHT1*;*3-RNAi* and *SiPHT1*;*4-RNAi* lines showed 26% and 41% reduction respectively for total P while the Pi contents were reduced to 82% in *SiPHT1*;*3-RNAi* line and to 90% reduction in *SiPHT1*;*4-RNAi* line (Fig. 6C).

## Discussion

Previous work documented the expression profile of the 12 members of the PHT1 phosphate transporter family of foxtail millet^8^. In order to study the *in vivo* function of this family a novel direct regeneration protocol for foxtail millet using shoot apex explants was developed and combined with *Agrobacterium*-mediated transformation to generate RNAi lines in which 3 PHT1 transporters *SiPHT1*;*2*, *SiPHT1*;*3* and *SiPHT1*;*4* were individually downregulated and the impact on plant growth and phosphate content analysed. Additional evidence for functional activity of SiPHT1;1, SiPHT1;2 SiPHT1;3 SiPHT1;7 and SiPHT1;8 was obtained through heterologous expression and ability to complement a *Δpho84* mutant of *S*. *cerevisiae*.

On 1 mM Pi, all *S*. *cerevisiae* strains grow as Pi can be taken up by low affinity transporters PHO87 and PHO90, the latter being the most important under high Pi conditions^16^. On low (0.2 mM) Pi, growth becomes dependent upon expression of a functional transporter with *k*_m_ in the micromolar range since mutants lacking the high affinity transport system comprising PHO84 and PHO89 show growth defects below 250 µM Pi^16^. PHO84 is the principal high affinity phosphate transporter in *S*. *cerevsiae* and complementation of *S*. *cerevisae pho84* mutants is a widely used method of testing functionality of plant phosphate transporters belonging to the PHT1 family^17–19^. Based on this test all but SiPHT1;4 could clearly rescue growth to some extent on 0.2 mM Pi and so encode phosphate transporters capable of scavenging Pi at micromolar concentrations. However heterologous expression is not an ideal system for detailed characterisation, as expression levels and efficiency of targeting to the plasma membrane will determine the amount of protein and therefore the amount of transport activity (*V*_max_). Indeed SiPHT1;4 must encode a functional transporter since its down regulation *in planta* resulted in reduced leaf and root phosphate content (Figs 5–7), so whether lack of observed complementation is due to expression/targeting issues or SiPHT1;4 being a low affinity transporter remains to be established. In contrast SiPHT1;2 clearly supported growth even on Pi concentrations as low as 10 µM (Fig. 1C) at least as well if not better than ScPHO84 which has a reported *K*_m_ in the low micromolar range.

The concept of separate low and high affinity transporters has been questioned recently as analysis of quadruple and quintuple PHT1 mutants in Arabidopsis showed progressive reduction of *V*_max_ but not *K*_m_, and both high and low affinity up take was observable in all the mutant lines^20^ suggesting that some transporters may show dual affinity. These authors argued that heterologous systems may not capture the natural regulatory processes, necessitating *in planta* studies to understand the roles of these transporters within an appropriate physiological context. To this end a novel direct regeneration and transformation protocol was developed to allow recovery of foxtail millet plants in which individual PHT1 transporters were downregulated.

Development of an efficient plant regeneration system seems to be essential for the *Agrobacterium*-mediated transformation and recovery of stable transgenic plants in cereals^21^. Millets are generally considered as recalcitrant for *in vitro* plant regeneration and transformation studies and have received less attention for these studies during past 2 decades when compared to other cereals^12^. Only a few plant tissue culture studies have been reported in foxtail millet^22^. In all these studies, the plants were regenerated through an intervening callus phase and the direct regeneration of foxtail millet has not been described. In this paper we report an efficient protocol for direct plant regeneration from shoot apex explants of foxtail millet with the use of two simple media containing only one phytohormone and minimal subculturing. Direct plant regeneration seems to be an effective approach to minimize somaclonal variation and results in minimal effort for subculturing cycles^23^.

Utilizing the direct regeneration procedure described we also developed a simple and efficient *Agrobacterium*-mediated transformation of foxtail millet. *Agrobacterium*-mediated transformation has been considered as a simple and cost-effective tool for the development of transgenic plants and functional genomic studies. Only a few reports are available for the *Agrobacterium*-mediated transformation of millets^22^. The first study on *Agrobacterium*-mediated transformation of foxtail millet was reported in 2005^13^ using immature inflorescence as explants with the transformation frequency of 6.6%. The same protocol was utilized to study the function of a pollen-specific gene *Si401* in foxtail millet^15^. Wang *et al*.^14^ further optimized the regeneration conditions following the *Agrobacterium*-mediated transformation of foxtail millet. However, in all these reports immature inflorescence was used as initial explants and callus mediated regeneration was adopted to recover the transgenic plants. The use of immature inflorescence as initial explants may pose some technical difficulties as these explants are not readily available around the year and needs pre-culture of plants. Further, callus mediated regeneration is a time consuming process and may also induce somaclonal variations. Therefore, the combination of an efficient *Agrobacterium*-mediated transformation of readily available (shoot apex) explants followed by direct regeneration is a significant advance on current methodology. Shoot apex seems to be an excellent source of explant for regeneration and transformation studies in cereals^24^. We have also previously reported an *Agrobacterium*-mediated transformation system for finger millet using shoot apex explants^25^. In this study following such a strategy we have achieved a transformation frequency of >9% which is higher than previous reports. The direct regeneration protocol reported for foxtail millet in this study should lay the foundation for designing similar protocols for other millets and related bio-energy crops like switchgrass. *Agrobacterium*-mediated transformation system has been well established in switchgrass and many important genes were studied in transgenic plants (reviewed in^26^).

Production of RNAi lines for *SiPHT1*;*2*, *SiPHT1*;*3* and *SiPHT 1*;*4* allowed investigation of their function *in planta*. Despite *SiPHT1*;*2* having overlapping expression patterns with *SiPHT1*;*3* and *SiPHT1*;*4*, single knock down lines for all 3 genes have clear phenotypes in both root and shoot with respect to growth and tissue Pi concentration, demonstrating that they are not functionally redundant (Figs 5, 6 and 7). This contrasts with results from Arabidopsis where loss of *PHT1*;*4* results in a 40% decrease in Pi absorption but only modest changes in internal root Pi^27,28^ and significant phenotypic changes were only seen in double *pht1*;*1Δpht1*;*4Δ* mutants^28^.

In rice *OsPHT1*;*8* is expressed in a wide range of tissues and is not induced by low Pi^29^ as is also the case or *SiPHT1*;*2* its close neighbour on the phylogenetic tree^8^. RNAi knock down of *OsPHT1*;*8* resulted in reduced shoot and root biomass under both high and low phosphate, reduced phosphate uptake and distribution, and reduced phosphate content^29^. Like *SiPHT1*;*2*, *OsPHT1*;*8* could complement a *S*. *cerevisiae pho84* mutant for growth on low Pi^29^. OsPHT1;8 is implicated in redistribution of Pi from source to sink^30^ and it is noteworthy that *SiPHT1*;*2* RNAi lines have strongly reduced leaf Pi (Figs 5 and 7). It will be interesting to determine if *SiPHT1*;*2* has a role in P allocation to reproductive tissues as *OsPHT1*;*8* does^30^.

*SiPHT1*;*3* is normally expressed in leaf with a suggestion of upregulation in older leaf on P starvation^8^. *SiPHT1*;*3* groups with *OsPHT1*;*5* and *OsPHT1*;*4* within subfamily IV of the PHT1 transporters^8,31^. *OsPHT1*;*5* does not appear to have been characterised in detail. *OsPHT1*;*4* is expressed primarily in root and embryo^32^ but it shows significant upregulation in shoots of older plants on P deprivation and can complement a *S*. *cerevisiae pho84* mutant^33^ as could *SiPHT1*;*3* in this study. Down regulation of *SiPHT1*;*3* resulted in reduced root and leaf phosphate under both low and high phosphate compared to wild type and reduced plant stature (Figs 5, 6 and 7). This is in contrast to the *OsPHT1*;*4* RNAi mutants which did not show significant changes in height or root length or in root or shoot phosphate under either high or low phosphate regimes^33^.

*SiPHT1*;*4* clusters with *OsPHT1*;*1* and *OsPHT1*;*2* in subfamily IV of the PHT1 transporters^8,31^. *OsPHT1*;*1* is constitutively expressed independent of P supply^34^. *SiPHT1*;*4* is predominantly expressed in root^8^ and shows low Pi dependent up regulation in older leaf therefore these two genes are probably not orthologous. *OsPHT1*;*2* is strongly induced by low P in roots (especially the stele and lateral roots but not epidermal and cortical cells) and only weakly expressed in leaf^17^. Like *SiPHT1*;*4* it could not complement a *pho84* mutant but showed low affinity mM range Pi uptake activity when expressed in Xenopus oocytes^17^. Knockdown of *OsPHT1*;*2* decreased transport of Pi to the shoot which was reflected in reduced P concentration in shoots^17^, a phenotype also seen in the current study.

Downregulation of *SiPHT1*;*4* resulted in the normally leaf expressed *SiPHT1*;*3* being upregulated in root samples (Fig. 7A). This effect appeared to be a specific consequence of loss of *SiPHT1*;*4* since no change in expression of *SiPHT1*;*3* was seen in the *SiPHT1*;*2 RNAi* plants. This upregulation is also not likely to reflect reduction in intracellular Pi since the reduction is as great or greater in *SiPHT1*;*2 RNAi* plants but perhaps a perturbation of a systemic signalling pathway. Interestingly several PHT1 transporters in *Arabidopsis* were shown to be systemically regulated in a split root experiment^35^. Hormones, sugars and microRNAs^36^ are all implicated and interconnected in systemic Pi signalling^37^.

Root architecture changes have been proposed to be a function of external Pi concentration^37–39^. However Pi starved roots show increased sensitivity to auxin due to upregulation of TIR1 promoting lateral root formation^40^. Here all 3 RNAi lines showed an exaggerated response to low Pi especially with induction of more lateral roots and root hairs. In contrast RNAi of *OsPHT1*;*1* resulted in no difference in root hair density and a reduction in root hair length in low Pi conditions compared to wild type plants^34^. In Arabidopsis, downregulation of transporters *AtPHT1*;*8* and *AtPHT1*;*9* produced a similar response of inhibition of primary root length with proliferation of lateral roots and root hairs under low Pi^19^ but contrasting results have also been obtained^41^ which may reflect complex interactions between phosphate and metal ion homeostasis^37,41^.

In summary we established a novel direct regeneration procedure for foxtail millet using readily available shoot apex explants and combined with *Agrobacterium*-mediated transformation system and characterised RNAi lines for 3 members of the PHT1 gene family. All 3 members showed phenotypes on both high and low Pi demonstrating non-redundancy of function. More detailed characterisation of these RNAi lines and higher resolution study of the expression should shed more light on their roles.

## Materials and Methods

### Plasmid construction

#### Plasmid construction for yeast complementation experiment

The coding sequences of PHT1 transporters SiPHT1;1, 1;2, 1;3, 1; 4, 1;7 and 1;8 were PCR amplified from genomic DNA (no introns present for these genes) and cloned into pDD-GFP-2^42^ using *Spe*I/*Age*I sites to generate C-terminal GFP tagged versions as PHO84-GFP had previously been shown to be functional^43^. The coding sequence of PHO84 of *Saccharomyces cerevisiae* (*ScPHO84*) was amplified from the genomic DNA of *S*. *cerevisiae* and cloned into pDDGFP-2 using *Spe*I/*Xma*I sites. All clones were confirmed by sequencing. The details of cloning primers are given in Supplementary Table S1. All these clones were confirmed by Sanger sequencing (Source Bioscience, UK) before moving onto the yeast complementation experiments. The plasmid maps of these clones were constructed using Vector NTI software (Life Technologies, NY, USA) and are included as Supplementary Figures S5 to S12.

#### Plasmid construction for RNA interference (RNAi) experiment

Plasmid pFGC1008 obtained from Arabidopsis Biological Resource Centre, USA (ABRC stock number CD3–446) was used to create the *S*. *italica PHT1-RNAi* (*SiPHT1-RNAi*) plasmids. The plasmid contained two pairs of unique restriction sites (*Asc*I/*Swa*I and *Bam*HI/*Spe*I) flanking a 335 base pair *GUS* fragment that serves to separate two components of inverted repeat. Regions of around 200 bp that are unique to each gene were identified by sequence alignment of *SiPHT1*;*2*, *SiPHT1*;*3* and *SiPHT1*;*4* and checked by BLAST to avoid the downregulation of any other isoform of SiPHT1 transporters. The *3′ UTR* regions were used for downregulating transporters SiPHT1;2 and 4 and an internal coding sequence was used for transporter SiPHT1;3 (Supplementary Figure S13). For cloning, primers were designed (Supplementary Table S2) with both pairs of restriction sites. In the first step of cloning, the amplified target was digested with *Asc*I/*Swa*I and ligated into the *Asc*I/*Swa*I-cleaved vector. This plasmid was used as a template for a second cloning with which to complete the inverted repeat construct. The same PCR product used for first digestion was used but this time digested with *Bam*HI/*Spe*I and inserted into the *Bam*HI/*Spe*I sites of the template plasmid and confirmed by sequencing. The plasmid maps of these clones were constructed using Vector NTI software (Life Technologies, NY, USA) and are included as Supplementary Figures (Supplementary Figures S1 to S3). The plasmids were separately mobilized into *Agrobacterium* strain LBA4404 using freeze thaw method^44^ and used for the transformation of foxtail millet.

### Yeast complementation assay

For yeast complementation of *S*. *cerevisiae* PHO84 mutant (*Δpho8*; Matα; his3*Δ*1; leu2*Δ*0; met15*Δ*0; ura3*Δ*0; YML123c::kanMX4) purchased from Euroscarf, Germany (http://web.uni-frankfurt.de/fb15/mikro/euroscarf/index.html). All the clones used in the assay were maintained on yeast nitrogen base (YNB) minus Uracil (-URA) plates with raffinose as carbon source. The expression of transporter was induced by the addition of galactose. The assay was performed with YNB without phosphate (Formedium, UK) and -URA broth. Pi was added to the specified concentration from 0.1 M KH_2_ PO_4_ stock. Acid washed glassware was used throughout. The liquid cultures were initiated with 3 ml YNB -URA broth containing glucose and 1 mM Pi. Single colonies were inoculated and grown overnight at 28 °C, 200 rpm. The cells were collected by centrifugation and washed with 5 ml sterile distilled water and finally diluted to 3 ml sterile distilled water. The complementation assay was performed in 30 mL YNB -URA broth cultures containing galactose as carbon source and containing 1 mM or 0.2 mM Pi with the inoculum adjusted to have the starting OD_600_ of 0.05 for all the cultures. The cultures were grown at 28 °C in an orbital shaker with 200 rpm.

### Plant tissue culture

The *S*. *italica* genotype ‘Maxima’ (Acc.No: Bs 3875)^8^ was used for the tissue culture and transformation studies. The seeds were surface sterilized and inoculated in Murashige and Skoog (MS;^45^ basal medium for germination (Hi-Media Mumbai, India). After 3 days of incubation, the shoot apex (4–8 mm in length) were excised inside the laminar flow under aseptic conditions and used for shoot induction. The shoot apexes were inoculated on multiple shoot induction medium (SIM) containing MS basal salts supplemented with different concentration of (0.5, 1.0, 1.5 or 2.0 mg/l) BAP, TDZ or KN (Hi-Media, Mumbai, India). The cultures were incubated in light for multiple shoot induction. The shoot clumps containing multiple shoots were transferred onto the shoot elongation medium (SEM) containing MS basal salts alone devoid of any plant growth regulators for elongation of multiple shoots. The shoots were rooted on the SEM after 4 weeks on incubation. The plantlets were separated, roots were washed with sterile water to remove the medium and transferred onto the paper cups containing sterile vermiculite and watered with diluted (10 time) MS basal medium and covered with polythene bags for 2 weeks. The plants were then transferred to the glass house and grown to maturity.

### *Agrobacterium*-mediated transformation of foxtail millet with RNAi plasmids

The shoot apex explants of foxtail millet were used for the transformation with *Agrobacterium* strain LBA4404 carrying 3 RNAi vectors (*pFGC-SiPHT1*;*2*, *pFGC-SiPHT1*;*3* or *pFGC-SiPHT1*;*4*). The stepwise protocol for *Agrobacterium*-mediated transformation is outlined in Fig. 3. On first day 1 (evening), a starter culture of *Agrobacterium* with plasmid was initiated by inoculation of a single colony of LBA4404 containing the RNAi vector into 3 ml yeast extract peptone (YEP) supplemented with 50 mg/L streptomycin, 35 mg/L chloramphenicol and 10 mg/L rifampicin. The cultures were incubated on a shaker for overnight at 28 °C and grown to OD_600_ = 0.8 to 1.0. On the second day, 100–200 µl of overnight grown starter culture was transferred to 30 ml of YEP (with all 3 antibiotics mentioned above for selection), 100 µM acetosyringone (AS) was added and the culture grown on the shaking incubator overnight to get an OD_600_ of 0.6–0.8. On the third day, the bacterial suspension was spun at 10,000 rpm (Eppendorf Centrifuge, Model-5810R, Rotor FA-45–6–30) at 4 °C for 10 min and the pellet was re-suspended in liquid SIM (MS + 0.5 mg/L BAP) to obtain a final OD_600_ = 0.6–0.8. Then 100 µM acetosyringone (AS) was added. The bacterial suspension was transferred into a sterile 50 ml glass beaker.

#### Infection of explants

Three days old shoot apex explants were excised and transferred into the 50 ml beaker containing bacterial suspension (40–50 explants/beaker). The beaker was incubated in orbital shaker at 28 °C for 10–15 min at 80 rpm. The explants were then transferred onto a sterile Whatman No.1 filter paper to remove the excess moisture (around 5 min).

#### Co-cultivation

Infected callus was transferred onto the sterile Whatman No.1 filter paper placed over the co-cultivation medium containing MS + 0.5 mg/L BAP and 100 µM AS. The cultures were incubated at 28 ± 2 °C for 3 days in dark. This method of co-cultivation helps to control the overgrowth of *Agrobacterium* by reducing the moisture and permits better plant recovery on selection^25^.

#### Selection and regeneration

After 3 days of co-cultivation, the explants were sub-cultured onto the selection medium (SIM containing 25 mg/l hygromycin and 250 mg/l cefotaxime) and incubated at 25 ± 1 °C in light with light intensity of 50 µmol m^−2^ s^−1^ photosynthetic photon flux density (PPFD). The cultures were checked regularly for cell death, contamination (if any) and induction of shoots. Any dead explants were removed from the medium to prevent the release of phenolics into the medium. The explants were sub-cultured once every 2 weeks into the selection medium. The hygromycin resistant shoots were transferred onto the SEM containing MS basal salts and 25 mg/l hygromycin and 250 mg/l cefotaxime for the recovery of transformed plantlets. The rooted plants were separated, roots were washed with sterile water to remove the medium and transferred onto the paper cups containing sterile vermiculite and watered with 10x diluted MS basal medium, hardened and moved to the greenhouse and grown to maturity as mentioned in the tissue culture section above. The seeds (T1 progeny) from primary transformants (T0) were germinated on MS basal medium containing 25 mg/L hygromycin. The seeds which germinated and established were transplanted in to the green house for further assays.

### Confirmation of transformation by PCR

Genomic DNA was isolated from hygromycin resistant plants (primary transformants, T0)^46^ and amplified with *hpt*II specific primers (Forward 5′GCTCCATACAAGCCAACCAC 3′; Reverse 5′CGAAAAGTTCGACAGCGTCTC3′). The PCR was performed in 25 µl reaction mixture containing 100 ng genomic DNA, 2.5 mM MgCl_2_, 0.25 mM dNTPs, 200 nM each of forward and reverse primers and 1 U *Taq* DNA Polymerase (Genet Bio, Daejeon, Korea) in an Eppendorf thermal cycler (Eppendorf Gradient Thermal Cycler, Germany) with an initial denaturation at 95 °C for 5 min followed by 35 cycles of 30 s denaturation at 95 °C, 30 s annealing at 61 °C and 1 min extension at 72 °C with a final extension at 72 °C for 10 min.

### Gene expression analysis using RT-PCR

Gene expression in wild type plants and T1 RNAi lines were analysed by RT-PCR based on the method described previously^8^. The details of primers used are listed in Supplementary Table S3. The PCR products were separated on 10% polyacrylamide gels as described previously^8^.

### Plant growth experiments and assay of phosphate contents

Inorganic phosphate content was analysed in 4 weeks old seedlings of T1 RNAi lines grown in perlite and supplied with nutrient solution containing 10 µM Pi^8^. The length of shoot and root and dry weights of root and shoot were determined in these seedlings. In order to dictate the more tightly controlled supply of external Pi, T1 plants of RNAi lines were also grown on hydroponics under 10 and 300 µM Pi conditions as mentioned in the previous report^8^, total and inorganic P contents were assayed using the protocol described by previously^47^.

The shoot length, root length, lateral root density and root hair density were analysed in 4 week old seedlings of T1 RNAi lines grown under low Pi (10 µM) in hydroponics. In later root density analysis, the roots were scanned using the scanner (HP Scanjet 200). The lateral root density were counted using the scanned images with the help of RootNav software^48^. The root hair density measurements were performed according to^49^ with some modifications. About 5 cm from the primary root cap was chosen for root hair analysis. The images were captured with a Leica Stereo Microscope (Wetzlar, Germany). The roots were placed on a stage micrometer with a scale (10 µm) and observed in Stereo Microscope (10x magnification). The image was then captured with a digital camera (Cannon Coolpix, Cannon). The root hair density was counted using ImageJ scientific software^50^.

### Statistical analysis

The experiments were conducted in a randomized block design. The number of replicates and repeats for each experiment is indicated in the figure legends. Mean values were calculated and the results were analysed on SPSS 16.0 (SPSS Inc., Chicago, IL, USA) using a *t*-test at the 1% level.

### Data availability statement

All data generated or analysed during this study are included in this published article (and its Supplementary Information files).

## Electronic supplementary material

Supplementary Information
