# Supplementary material for: Functional characterization of the PHT1 family transporters of foxtail millet with development of a novel Agrobacterium-mediated transformation procedure
Source: Sci Rep. 2017 Oct 25;7:14064. doi: 10.1038/s41598-017-14447-0 (PMC5656669; doi:10.1038/s41598-017-14447-0)
Supplement: Supplementary file 1 — Supplementary Information [file 41598_2017_14447_MOESM1_ESM.pdf]

SUPPLEMENTARY INFROMATION

Functional characterization of the PHT1 family transporters of foxtail millet with development of a novel *Agrobacterium*-mediated transformation procedure

S. Antony Ceasar [1, 2, *], Alison Baker[1], S. Ignacimuthu[2]

1. Centre for Plant Sciences and School of Molecular and Cellular Biology, Faculty of Biological Sciences, University of Leeds, Leeds LS2 9JT, UK

2. Division of Plant Biotechnology, Entomology Research Institute, Loyola College, Chennai 600034, India

***Corresponding author:** antony_sm2003@yahoo.co.in

List of contents

- **Table S1:** Details of cloning primers used to clone SiPHT1 transporters into pDDGFP-2 plasmid for yeast complementation assay experiments
- **Table S2:** Details of cloning primers used to clone SiPHT1 transporter fragments into ppFGC1008 plasmid for down regulation of these genes
- **Table S3.** Details of primers used for RT-PCR analysis of T1 progenies RNAi transgenic lines
- **Figure S1.** Map of the plasmid *pFGC-SiPHT1;2-3'UTR-RNAi*
- **Figure S2.** Map of the plasmid *pFGC-SiPHT1;3-RNAi*
- **Figure S3.** Map of the plasmid *pFGC-SiPHT1;4-3'UTR-RNAi*
- **Figure S4.** Screening of foxtail millet for hygromycin sensitivity
- **Figure S5.** Map of the yeast expression vector *PDDGFP-2*
- **Figure S6.** Map of the plasmid *pDDGFP-SiPHT1;1*
- **Figure S7.** Map of the plasmid *pDDGFP-SiPHT1;2*
- **Figure S8.** Map of the plasmid *pDDGFP-SiPHT1;3*
- **Figure S9.** Map of the plasmid *pDDGFP-SiPHT1-4*
- **Figure S10.** Map of the plasmid *pDDGFP-SiPHT1-7*
- **Figure S11.** Map of the plasmid *pDDGFP-SiPHT1-8*
- **Figure S12.** Map of the plasmid *pDDGFP-PHO84*
- **Figure S13.** Details of gene sequences used for the development of RNAi plasmids for foxtail millet transporters SiPHT1;2, SiPHT1;3 and SiPHT1;4

Supplementary Table S1: Details of cloning primers used to clone SiPHT1 transporters into *pDDGFP-2* plasmid for yeast complementation assay experiments

**Name of the gene**	**Forward primer (5' to 3')**	**Reverse primer (5' to 3')**	**Tm**	**Product length**
SiPHT1;1	GATCACTAGTATGGCGAGGCAGGAGCGG *Spe*I	GGTTACCGGTCACCATTTCAAGTCCGGAAGGC *Age*I	63.46	1629
SiPHT1;2	GATCACTAGTATGGCGCGTGGGGGCGAC *Spe*I	GGTTACCGGTCACCATCTGGGTCTGGGACGG *Age*I	66.46	1623
SiPHT1;3	GATCACTAGTATGGCCCACGATCACAAGG *Spe*I	GGTTACCGGTTTCGCTCAAATTGGTCGGAAC *Age*I	59.77	1608
SiPHT1;4	GATCACTAGTATGGCGGGAGCTCAGCTC *Spe*I	GGTTACCGGTGACTCTGGCCGGAGCATC *Age*I	60.87	1581
SiPHT1;7	GATCACTAGTATGGCTGGCGGCGACCTG *Spe*I	GGTTACCGGTCACGGGCACGGTGCGGTTG *Age*I	66.32	1617
SiPHT1;8	GATCACTAGTATGGCGCGGGAGAAGCTGC *Spe*I	GGTTACCGGTCAGCGGTAGAATCTGGGAGTCG *Age*I	64.17	1623
PHO84	GATCACTAGTATGAGTTCCGTCAATAAAGATACT *Spe*I	GGTTCCCCGGGTGCTTCATGTTGAAGTTGAGATG *Xma*I	56.66	1761

Supplementary Table S2: Details of cloning primers used to clone SiPHT1 transporter fragments into *pFGC1008* plasmid for down regulation of these genes

Name of the gene	Forward primer (5' to 3')	Reverse primer (5' to 3')
SiPHT1;2	GC ACTAGT GGCGCGCC GGTGTAGTATGACCGTCCGTG<br>*Spe*I *Asc*I	GA GGATCC ATTTAAAT GCGTGGTATGTTCACCAATG<br>*Bam*HI *Swa*I
SiPHT1;3	GC ACTAGT GGCGCGCC CAAGACGGATGCCGGTTAC<br>*Spe*I *Asc*I	GA GGATCC ATTTAAAT GTCGGAACAGTCTGCTGGATG<br>*Bam*HI *Swa*I
SiPHT1;4	GC ACTAGT GGCGCGCC CAGAGTCTAGGGGCCTGCAG<br>*Spe*I *Asc*I	GA GGATCC ATTTAAAT GGGAATGCTGAAGTACAACAGC<br>*Bam*HI *Swa*I

Supplementary Table S3. Details of primers used for RT-PCR analysis of T1 progenies RNAi transgenic lines

Name of the gene	Forward primer (5’ to 3’)	Reverse primer (5’ to 3’)	Product length (bp)	Ann. Temp
SiPHT1;2	ACCAGGACAAGAGCAAGGTG	GGCACGAGGAACGTGAGTAT	115	60.22
SiPHT1;3	TGTCATCGGGTTCTTGTTCA	AATTGGTCGGAACAGTCTGC	128	60.10
SiPHT1;4	CAGAAGGAGATCCAGGACGA	CGATATCGAGCAGGAACCAC	145	60.48
Si-actin-2	ACGACCATGTTCCCTGGTATT	ATCGTACTCCGCCTTTGAGAT	183	59.0

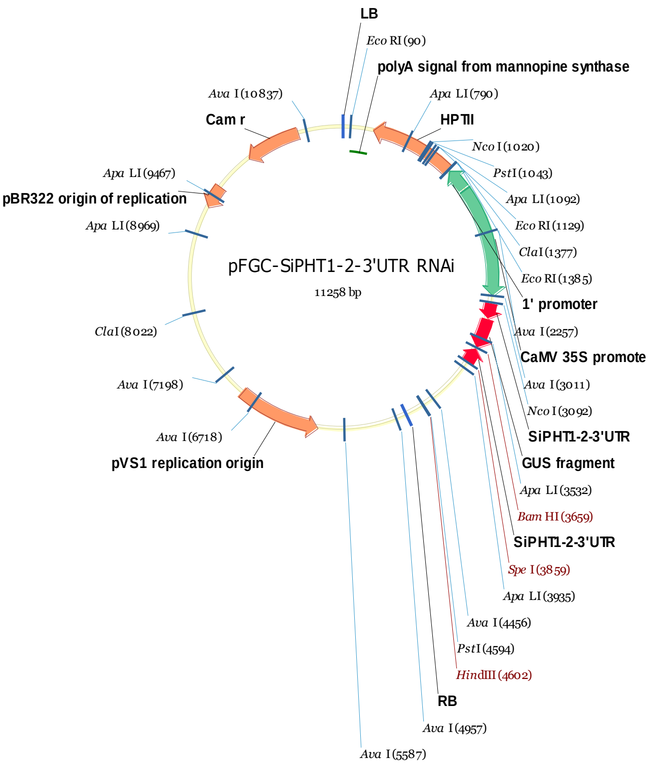

Supplementary Figure S1. Map of the plasmid *pFGC-SiPHT1;2-3'UTR-RNAi*.

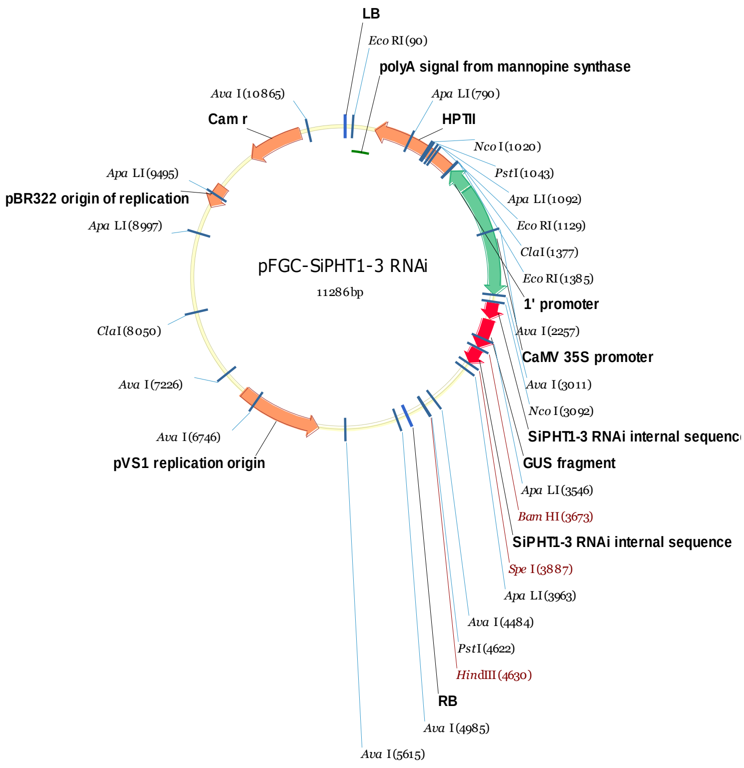

Supplementary Figure S2. Map of the plasmid *pFGC-SiPHT1;3-RNAi*.

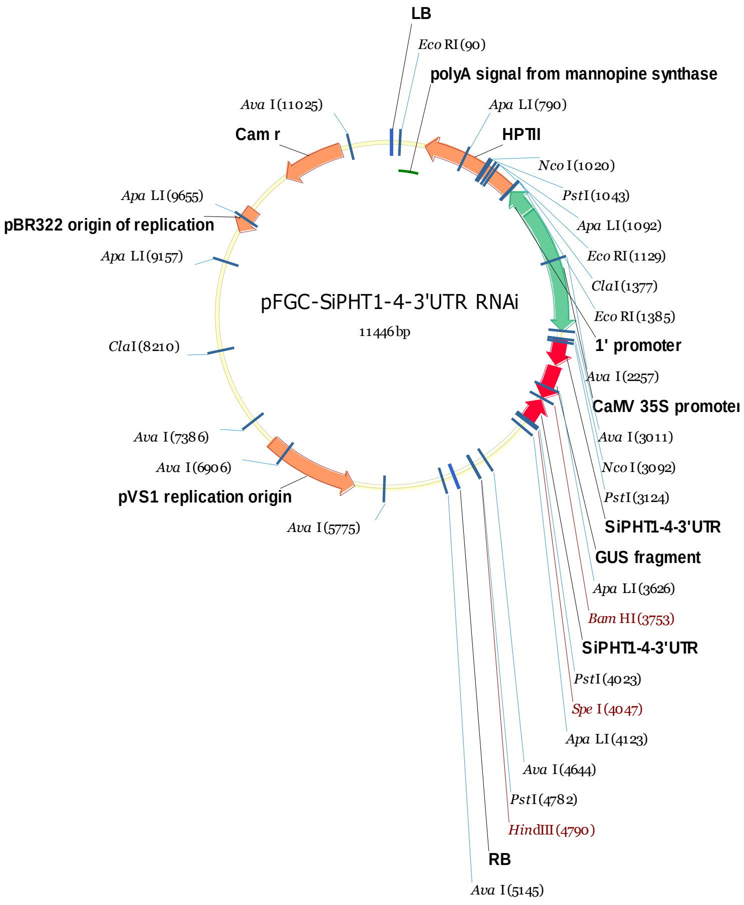

Supplementary Figure S3. Map of the plasmid *pFGC-SiPHT1;4-3'UTR-RNAi*.

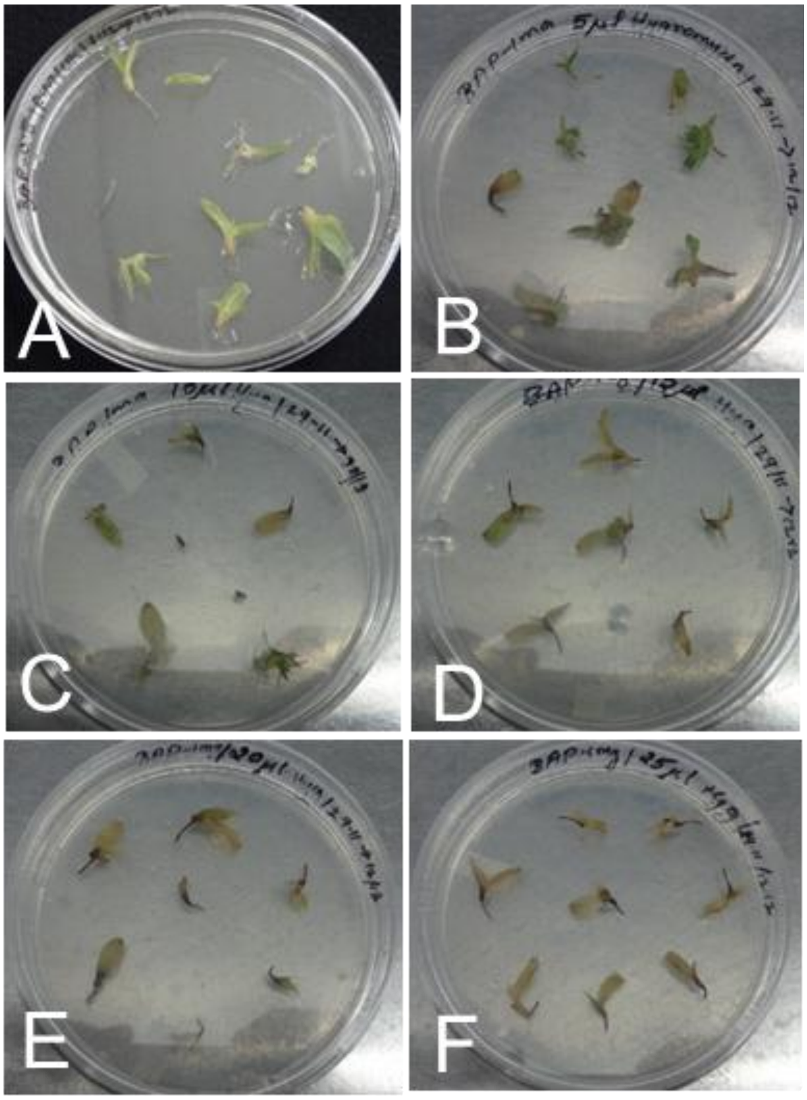

Supplementary Figure S4. Screening of foxtail millet for hygromycin sensitivity. The shoot apex explants were cultured on MS medium containing 0.5 mg/L BAP without hygromycin (A), and 0.5 mg/L BAP with 5.0 mg/L (B), 10.0 mg/L (C), 15.0 mg/L (D), 20.0 mg/L (E) and 25.0 mg/L (F) hygromycin. The response was noted one week after culture in the light. The explants cultured in the medium containing 20 mg/L hygromycin (E) or above (F) showed 100% mortality.

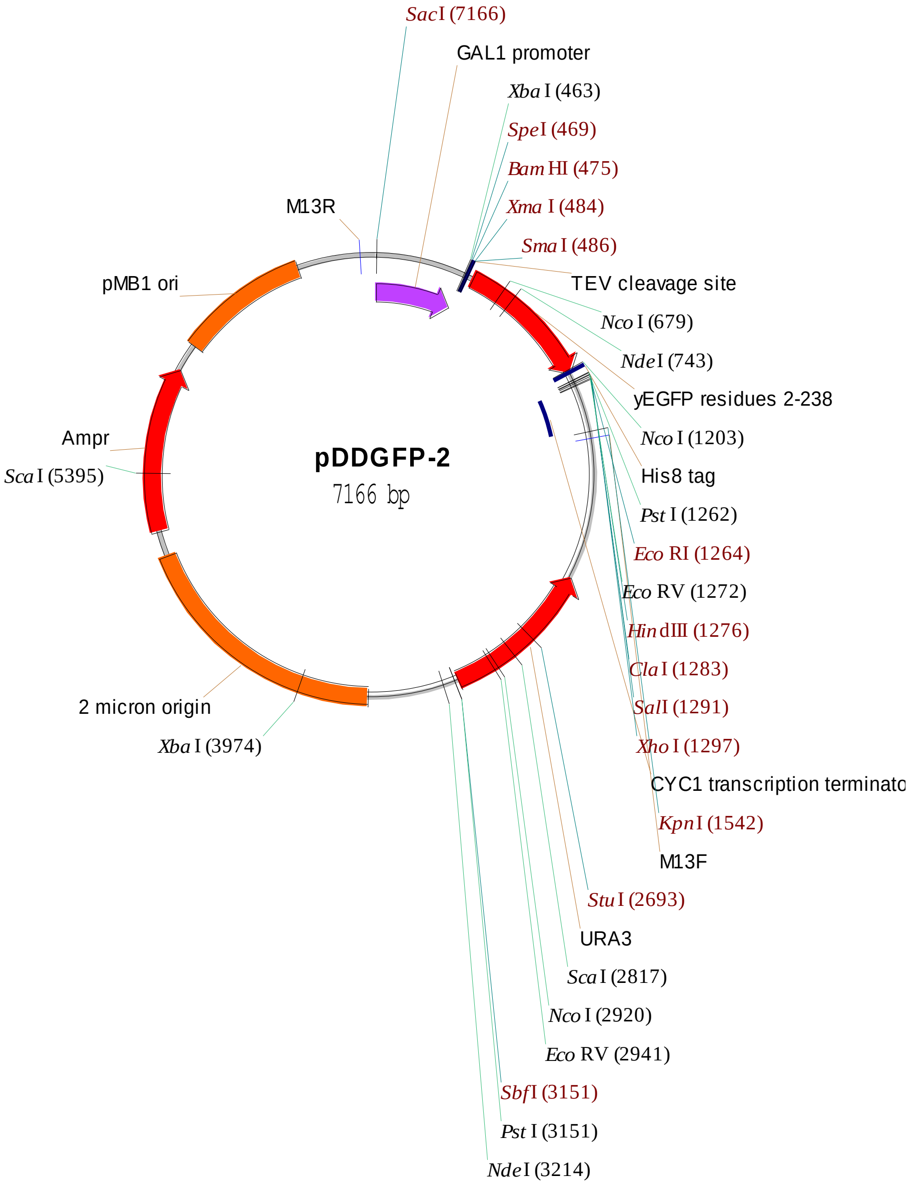

Supplementary Figure S5. Map of the yeast expression vector *PDDGFP-2* (parental plasmid) used for cloning coding sequences of SiPHT1 and PHO84 for yeast complementation assay. The transporters were cloned Gal1 promoter and YGFP regions using *Spe*I/*Age*I sites for SiPHT1 transporters and *Spe*I/*Xma*I sites for PHO84 transporter. The plasmid has Ampr region for bacterial selection and URA3 for yeast selection.

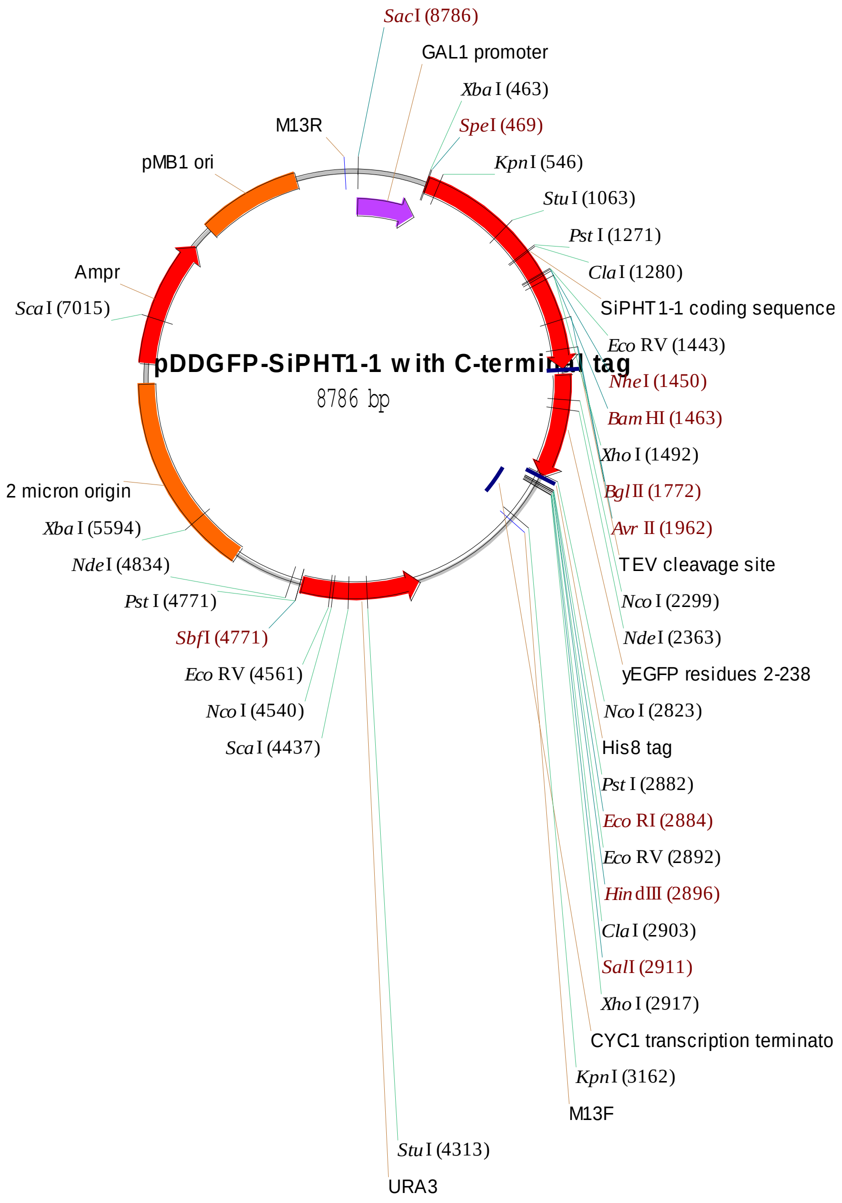

Supplementary Figure S6. Map of the plasmid *pDDGFP-SiPHT1;1*

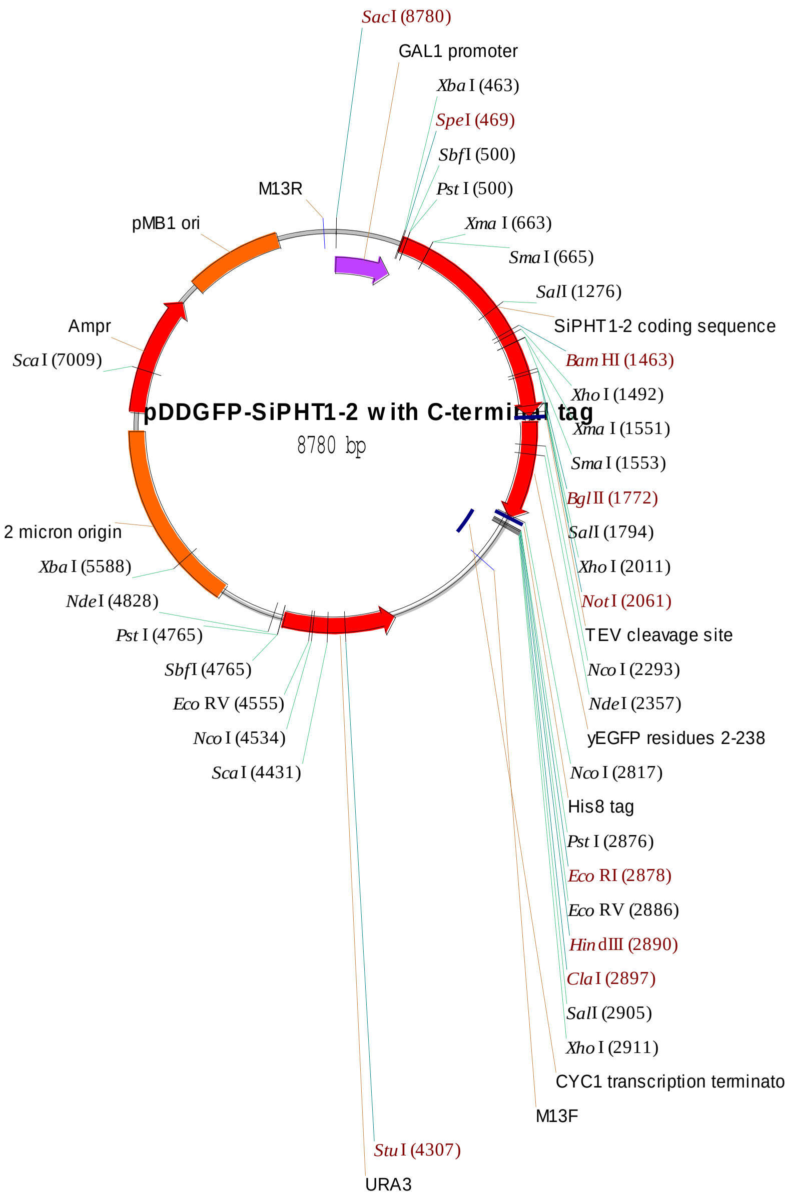

Supplementary Figure S7. Map of the plasmid *pDDGFP-SiPHT1;2*

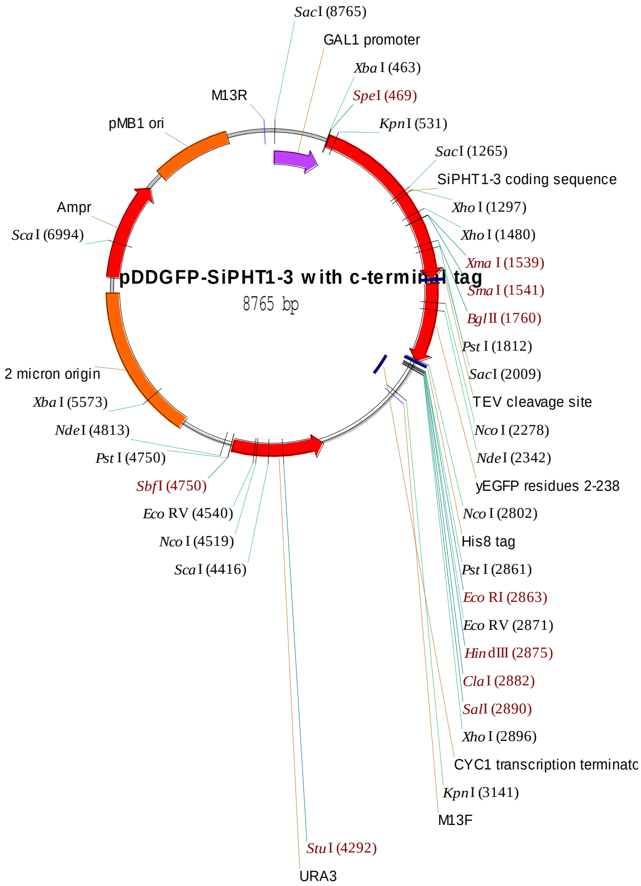

Supplementary Figure S8. Map of the plasmid *pDDGFP-SiPHT1;3*

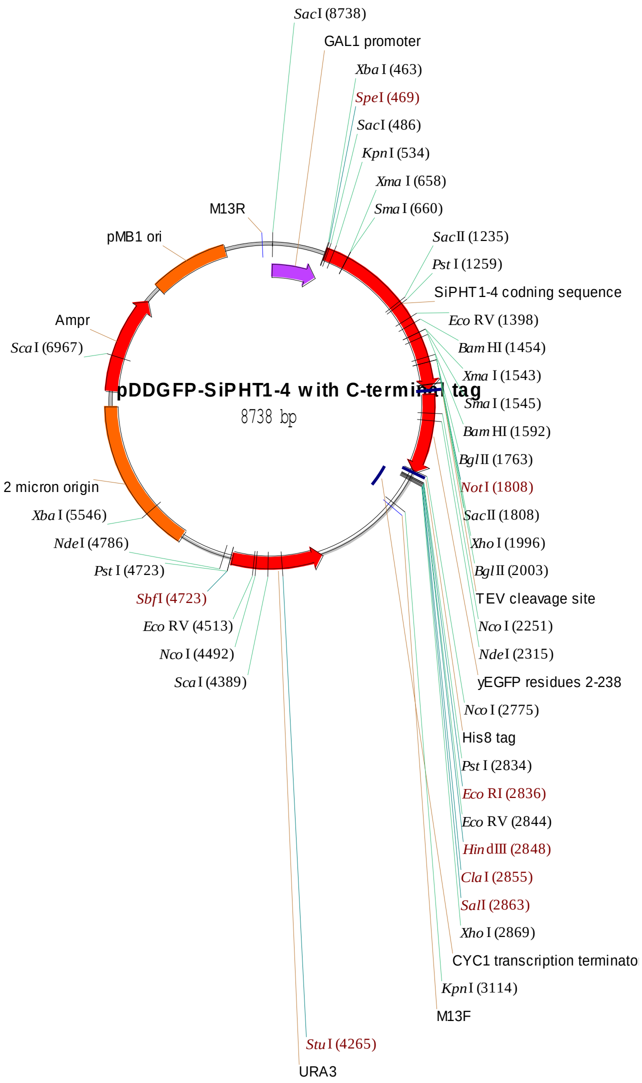

Supplementary Figure S9. Map of the plasmid *pDDGFP-SiPHT1-4*

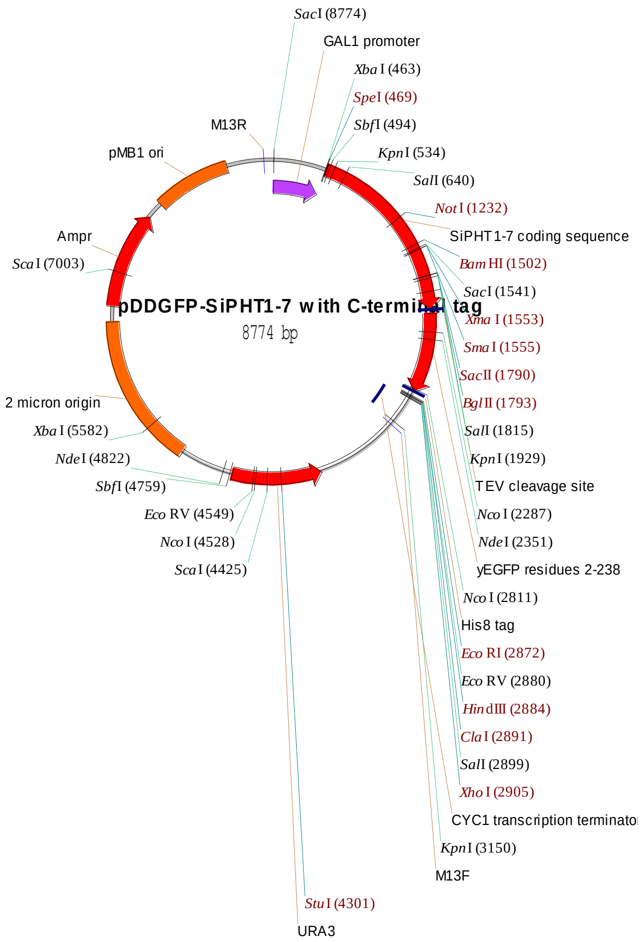

Supplementary Figure S10. Map of the plasmid *pDDGFP-SiPHT1-7*

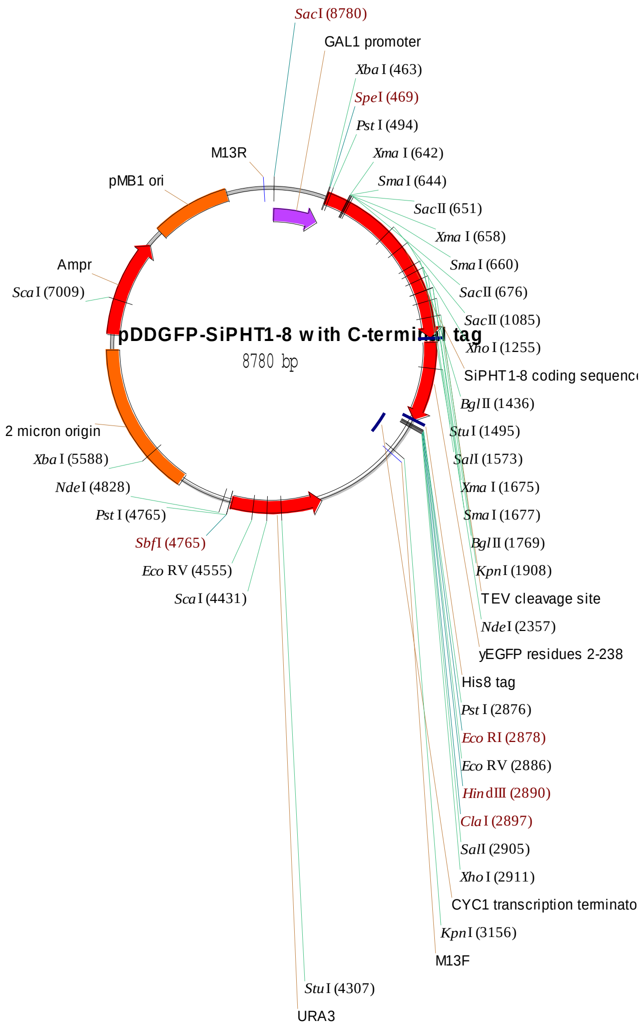

Supplementary Figure S11. Map of the plasmid *pDDGFP-SiPHT1-8*

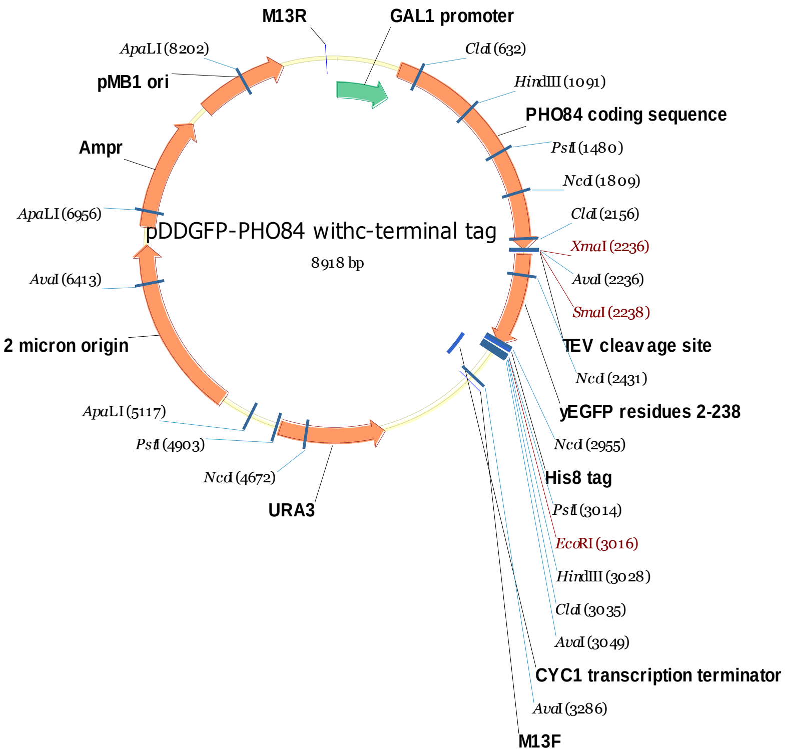

Supplementary Figure S12. Map of the plasmid *pDDGFP-PHO84*

SiPHT1;2 3′UTR sequence used for targeting through RNAi

GGTGTAGTATGACCGTCCGTGGTGATTGGTGATACGTGTAGGCCGGTTCACTTGTTTTCGTT
TTCCATGTAGAAAGTCAAACCTGCTGTTTCACATGGGCATCTGTTATTTTTATCTCTATATA
AAATATAAAAAAGAAAATATCAAGTACACAAATACATTGGTGAACATACCACGC

SiPHT1;3 coding sequence used for targeting through RNAi

CAAGACGGATGCCGGTTACCCGCCAGGCATCGGCGTGCGCAACTCACTGTTCATGCTCGCCG
GATGCAATGTCATCGGGTTCTTGTTCACGTTCCTTGTGCCGGAGTCCAAGGGAAAGTCGCTG
GAGGAGCTCTCCGGCGAGAACGACGAGGAGGCAGCACCTGGCCAGAGCATCCAGCAGACTGT
TCCGAC

SiPHT1;4 3′UTR sequence used for targeting through RNAi

CAGAGTCTAGGGGCCTGCAGCTCCCCCCACACACTTTCTGCGCGCGTGCCTACATGATGCAC
GGATGGTTTTCAGGTTCTGTTTGTATGCTTGACTGTGTCCTTGTGTGGTTTACATCATACTC
CTACCTTCCGATTTATTGCATTCTGTGTGATATTTGAAAATATTTTTGGGTAATTTCTCCAC
TGAGAATTTCGTTTGTGCTAGTGTTTGTGTCTGATTGACCGACATTCTTATCAATGAATAAA
AAGCTGTTGTACTTCAGCATTCCC

Supplementary Figure S13. Details of gene sequences used for the development of RNAi plasmids for foxtail millet transporters SiPHT1;2, SiPHT1;3 and SiPHT1;4. The binding regions of forward and reverse primers are indicated in blue and red respectively.
